# Fat or flat? The impact of dipole moment vectors on non-covalent interactions between aromatic tags and macromolecules

**DOI:** 10.1039/d5qi01546d

**Published:** 2025-10-21

**Authors:** Josef Holub, Adéla Jílková, Carina Lemke, Lorenzo Cianni, Petra Spiwoková, Martin Horn, Christian Breuer, Adrian Leontovyč, Jiří Brynda, Helena Mertlíková-Kaiserová, Marta Chanová, Fernanda dos Reis Rocho, Carlos A. Montanari, Nelly El-Sakkary, Conor R. Caffrey, Michael Gütschow, Drahomír Hnyk, Michael Mareš, Jindřich Fanfrlík

**Affiliations:** a Institute of Inorganic Chemistry of the Czech Academy of Sciences Husinec-Řež 250 68 Czech Republic hnyk@iic.cas.cz; b Institute of Organic Chemistry and Biochemistry of the Czech Academy of Sciences Flemingovo nám. 2 16610 Prague 6 Czech Republic mares@uochb.cas.cz fanfrlik@uochb.cas.cz; c Pharmaceutical Institute, Pharmaceutical & Medicinal Chemistry, University of Bonn An der Immenburg 4 53121 Bonn Germany guetschow@uni-bonn.de; d Medicinal and Biological Chemistry Group, São Carlos Institute of Chemistry, University of São Paulo Avenue Trabalhador Sancarlense 400 13566-590 São Carlos/SP Brazil; e Institute of Medicinal Microbiology, First Faculty of Medicine, Charles University and General University Hospital in Prague Studničkova 7 12800 Prague 2 Czech Republic; f Center for Discovery and Innovation in Parasitic Diseases, Skaggs School of Pharmacy and Pharmaceutical Sciences, University of California San Diego 9255 Pharmacy Lane MC0657 La Jolla CA 92093 USA

## Abstract

The *closo*-1,2-C_2_B_10_H_12_ carborane is a recognized 3D aromatic icosahedral building block, with an electron distribution governed by the outer hydridic BH and acidic CH vertices. We attached the carborane cage to a peptidomimetic scaffold to generate an active-site inhibitor of SmCB1, a protease drug target in the Schistosoma pathogen. The carborane-tagged compound exhibited superior inhibitor affinity and bioactivity compared to its conventional 2D aromatic phenyl analog. Quantum mechanical computations, based on the crystal structure of the protease–inhibitor complex, revealed that the carborane tag contributed to inhibitor binding not only through nonpolar interactions but also *via* a key hydrogen bond between its CH vertex and a negatively charged residue in the binding site. This interaction, driven by the large dipole moment of the carborane cage, resulted in a more favorable energy contribution than that of the phenyl group in the 2D analog. The carborane pharmacophore boosted affinity for SmCB1 and conferred specific anti-schistosomal activity, highlighting its potential in protein ligand design.

## Abstract 2

The *closo*-1,2-C_2_B_10_H_12_ carborane is a recognized 3D aromatic icosahedral building block, with an electron distribution governed by the outer hydridic BH and acidic CH vertices.

## Graphical abstract

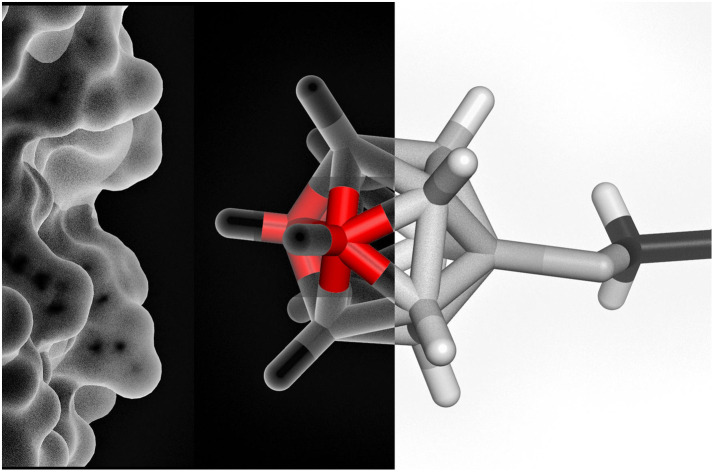

## Introduction

Boron has a range of applications across materials’ chemistry, catalysis and medicinal chemistry.^1–6^ Positioned next to carbon in the periodic table, it shares some similarities with carbon but also exhibits key differences. Specifically, boron chemistry is characterized by three-dimensional (3D) cage-like clusters stabilized by three-center, two-electron (3c–2e) bonds.^7,8^ This bonding arises because boron lacks sufficient valence electrons to form conventional two-center, two-electron (2c–2e) bonds connecting all atoms in a molecule. Clusters that have only triangular faces are designated by the prefix “*closo*” (from the Latin *clusus*, meaning “closed”), among them *closo*-B_12_H_12_^2−^ being the most stable example. Carboranes are a class of boron clusters in which one or more BH units are formally replaced by isoelectronic CH^+^ groups. Carborane chemistry is dominated by icosahedral *ortho*-carborane, *closo*-1,2-C_2_B_10_H_12_ (1), one of the most studied boron clusters.^9^ Occupying a volume comparable to that of a rotating phenyl ring due to the similarity in their body diagonals, carborane 1 represents a 3D analog of benzene. In contrast to benzene, the cage of 1 consists of the CH and BH vertices that are distinct in their properties. The BH vertices are hydridic and can form dihydrogen (diH) bonds—interactions between hydridic BH^*δ*−^ sites and the H^*δ*+^ atoms, *e.g.*, NH or OH groups of biomolecules.^10,11^ Additionally, BH vertices can also form nonpolar interactions with the hydrophobic regions of a protein, primarily through London dispersion forces.^11^ In contrast, the CH vertices of 1 are acidic and interact favorably with negatively charged sites.^12^ Unlike benzene, which has a zero dipole moment value, the electron distribution of 1 is dominated by a partial positive charge in the middle point of the C–C vector with the dipole moment value of 4.5 D.^13^ This value is comparable to that of strongly polar organic molecules such as acetonitrile (3.9 D) and nitrobenzene (4.3 D).

Featuring special physical and chemical properties, carborane 1 has the potential to serve as a novel pharmacophore for drug design and discovery.^14,15^ Indeed, carborane and its derivatives have been shown to be useful chemical entities in antitumor medicinal chemistry.^1,16^ The applications of carboranes and their derivatives in the field of antitumor research mainly include boron neutron capture therapy (BNCT), as BNCT/photodynamic therapy dual sensitizers, and as anticancer ligands.^16^ However, in comparison to benzene chemistry, this research area is still in its infancy, and selective tagging of carboranes to ligand scaffolds has been receiving increasing research attention.^1,16^

**Fig. 1:**
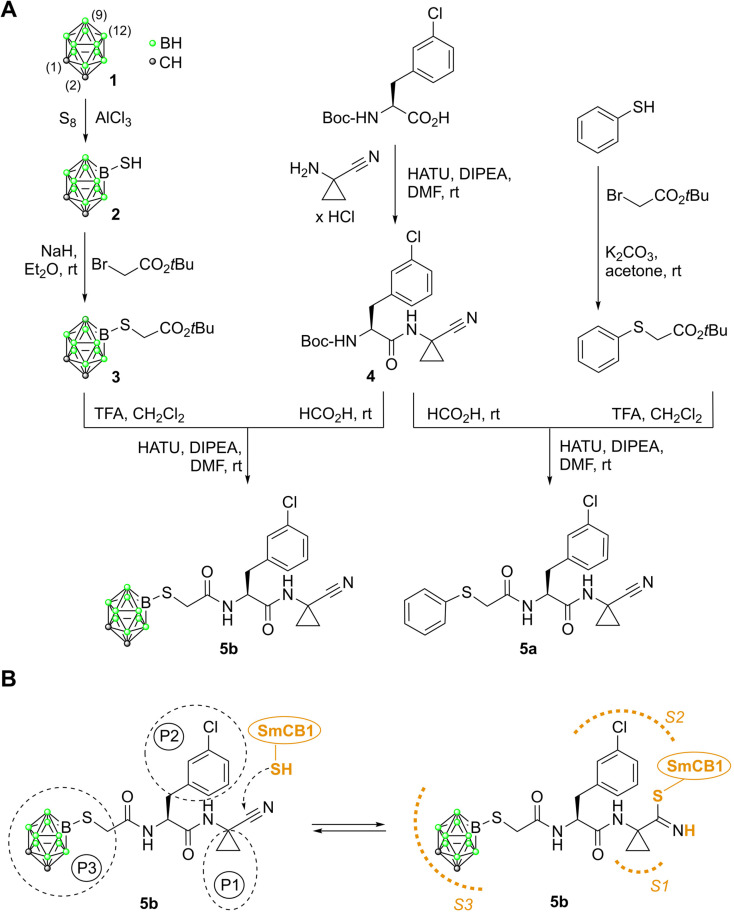
(A) The preparation of the carborane inhibitor 5b, alongside its phenyl analog 5a. Standard numbering for selected vertices of 1 is indicated by the numbers in brackets. (B) Mechanism of inhibitor binding illustrated with 5b: The reactive nitrile warhead forms a covalent reversible thioimidate bond with the thiol group of the catalytic cysteine in the *Schistosoma mansoni* cathepsin B-like protease, SmCB1. The inhibitor side chains (P1–P3) occupy the canonical subsites (S1–S3) of SmCB1.

In this study, we synthesized a carborane-tagged peptidomimetic ligand and examined its potency in terms of intermolecular interactions and bioactivity, compared to its conventional 2D aromatic phenyl analog. The connection between carborane 1 and the peptidomimetic scaffold was achieved *via* a -S-CH_2_-CO- linker (Fig. 1).^17^ We propose the adduct of the carborane 1 and the linker as a useful building block in medicinal chemistry. Carborane 1 was attached to the linker *via* a B(9)–S bond. Functionalization of the hydridic BH group on carborane 1 was specifically chosen to maintain the availability of both the CH and BH groups on the cage surface for potential noncovalent interactions with the active site of the protease. The B(9) position was selected for functionalization, as the BH vertices, located antipodally to the CH vertices—specifically B(9) or B(12) (Fig. 1)—are the most reactive and accessible sites for substitution under electrophilic conditions.^18^

The carborane cage 1 was incorporated into a peptidomimetic scaffold of an active site-directed inhibitor of the cysteine protease, SmCB1, which is a key protease responsible for digesting blood as a nutrient resource by the flatworm parasite *Schistosoma mansoni.*^19^ Inhibition of this cathepsin B-like protease, is a validated approach^20^ for the development of therapeutics against schistosomiasis,^21,22^ a neglected disease that infects >250 million people in the tropics and subtropics.^23^ Peptidomimetic inhibitors of SmCB1 with different reactive warheads, *e.g.*, vinyl sulfones or nitriles, are attractive anti-schistosomal agents, and novel derivatives are under development.^24–26^ These efforts are important as current treatment and control of schistosomiasis are precariously reliant on just one partially-effective drug, praziquantel.^27–29^

For the SmCB1 target, we demonstrate that inhibition potency and bioactivity against the parasite of the carborane-tagged peptidomimetic inhibitor are superior to its 2D aromatic phenyl analog. Quantum mechanical computations based on the crystal structure of the SmCB1–inhibitor complex revealed that this enhanced activity is primarily due to a favorable hydrogen bond between the CH vertex of the carborane cage and the negatively charged subsite of SmCB1. These findings highlight the carborane cage as a valuable pharmacophore with the potential to enhance functional properties *via* interactions driven by its particular steric and electrostatic features.

## Results and discussion

### Compound design and preparation

The synthesis of the inhibitors is summarized in Fig. 1 (see SI for experimental details). A nitrile warhead was used as part of the 1-aminocyclopropane-1-carbonitrile fragment at the P1 position.^30,31^ The rationale behind the selection of representatives of the chemotype of peptide nitriles was based on the following considerations. (i) Peptide nitriles possess a particularly small electrophilic warhead that does not interfere with the interaction of the binding sites of the protease with the corresponding amino acid residues of the inhibitor. The likewise established peptidyl vinyl sulfones, for example, have larger warheads. (ii) In contrast to carbanitriles, peptidic azanitriles, albeit highly potent,^26,32,33^ necessarily bear alkyl substituents at both nitrogens of the C-terminal aza-amino nitrile building block to prevent unwanted 5-*exo*-dig cyclizations.^34–36^ For the present design, however, it was preferred to maintain the CO–NH connection between the P2 and P1 substructures. (iii) Peptide nitriles constitute a successful class of peptidomimetic drugs, as can be exemplified by odanacatib that reached phase III clinical trials as a medication against osteoporosis and bone metastasis,^37^ and particularly by nirmatrelvir, an inhibitor of the main protease of SARS-CoV-2, that is approved to treat COVID-19.^38^

At the P2 position, we introduced 3-chlorophenylalanine, a particularly beneficial moiety to target parasite cysteine proteases.^39–41^ Carborane 1 was linked to the ligand scaffold *via* the B(9) position. Carboranethiol, *i.e.*, 9-SH-*closo*-1,2-C_2_B_10_H_11_ (2), was prepared from 1 by Lewis acid-assisted thiolation.^42^ Subsequent alkylation with *tert*-butyl bromoacetate yielded compound 3. In a parallel approach, benzenethiol was converted into *tert*-butyl 2-(phenylthio)acetate. Both *tert*-butyl 2-(phenylthio)acetate and 3 were then coupled to a dipeptide derivative, 4, affording the final products 5a and 5b.

### Inhibition of SmCB1 and other cysteine proteases, and *in vitro* anti-schistosomal activity

**Fig. 2:**
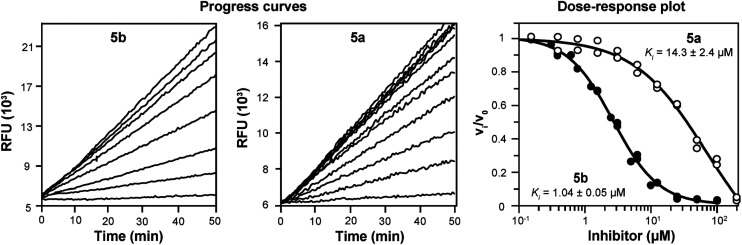
Inhibition kinetics of SmCB1 in the presence of the inhibitors 5b and 5a. The progress curves show the hydrolysis of the fluorogenic substrate Cbz-Phe-Arg-AMC (Cbz, benzyloxycarbonyl; AMC, 7-amino-4-methylcoumarin) by SmCB1 in the presence of increasing inhibitor concentrations. Linear progress curves obtained for 5a and 5b are characteristic of fast-binding inhibitors. In a dose–response plot, the derived steady-state reaction rates were plotted against inhibitor concentration, and the inhibition constants *K*_i_ were obtained.

The compound 5b with the carborane cage at the P3 position, its 2D aromatic phenyl analog 5a, and their precursors were evaluated as inhibitors of SmCB1, a cathepsin B-like protease target from the *S. mansoni* parasite, as well as its human homolog, cathepsin B (cat B), which is involved in lysosomal protein turnover in host tissues (Table 1). The inhibition constants *K*_i_ were determined using a kinetic inhibition assay with peptidyl substrates.^43^ Notably, 5b exhibited an order of magnitude stronger inhibition of SmCB1 (*K*_i_ of 1.0 μM) than its analog 5a (*K*_i_ of 14.3 μM; Table 1 and Fig. 2). This difference was also observed for cat B, although less pronounced (2.2 μM *vs.* 6.8 μM). The common short peptidic precursor of both inhibitors, compound 4 with the nitrile warhead, inhibited the parasite and human enzymes with lower potency (*K*_i_ of 27.1 and 15.5 μM, respectively). The three carborane precursors (1, 2 and 3) lacking the nitrile warhead were inactive against SmCB1 and cat B, except for the weak inhibition of cat B by 2. Overall, 5b, which included the carborane cage, was a more potent inhibitor of both enzymes than its analog, 5a, which contained the planar phenyl group. The incorporation of 3D aromatics resulted in a considerable improvement in affinity for SmCB1 (14-fold) compared to cat B (3-fold). Carborane tagging also provided a certain degree of selectivity for the parasite enzyme, which increased from 0.5 (5a) to 2.2 (5b; Table 1).

**Table 1:** Inhibition of SmCB1 and human cathepsin B (cat B) by the designed compounds and their precursors

Cpd.	*K*_i_ a (μM)		Selectivity
	SmCB1	Cat B	*K*_i_ (cat B)/*K*_i_ (SmCB1)
5b	1.0 ± 0.1	2.2 ± 0.1	2.2
5a	14.3 ± 2.4	6.8 ± 0.5	0.5
4	27.1 ± 3.2	15.5 ± 1.7	0.36
1	>100	>100	n.a.^b^
2	>100	43.8 ± 5.6	n.a.
3	>100	>100	n.a.

a The inhibition-constant (*K*_i_) values were determined using a kinetic activity assay with fluorogenic/chromogenic peptide substrates. The standard errors of triplicate measurements refer to non-linear regression.

b n.a., not applicable.

In addition, we investigated whether the replacement of a 2D aromatic with a 3D aromatic at the P3 position would improve the affinity for other cathepsin proteases (Table S1), namely, the cathepsin L-like cruzain from the protozoan parasite, *Trypanosoma cruzi*,^41,44^ and human cathepsins K, L and S.^32,45,46^ Again, compound 5b outperformed its phenyl analog in three of four cases. Only human cathepsin S was preferentially inhibited by 5a and even by the untagged precursor 4. The *K*_i_ values of 5b and 5a, screened against these proteases, showed less pronounced differences than the data generated for SmCB1.

Based on the enzymological data, it can be concluded that the carborane cage relative to the phenyl ring at the P3 position enhances the potency of inhibition of most cysteine-class cathepsin proteases, with a prominent contribution to SmCB1 inhibition. We speculate that the charge distribution and spatial properties of the S3 binding subsite in each target enzyme that interacts with the 3D aromatics account for the final affinity.

**Fig. 3:**
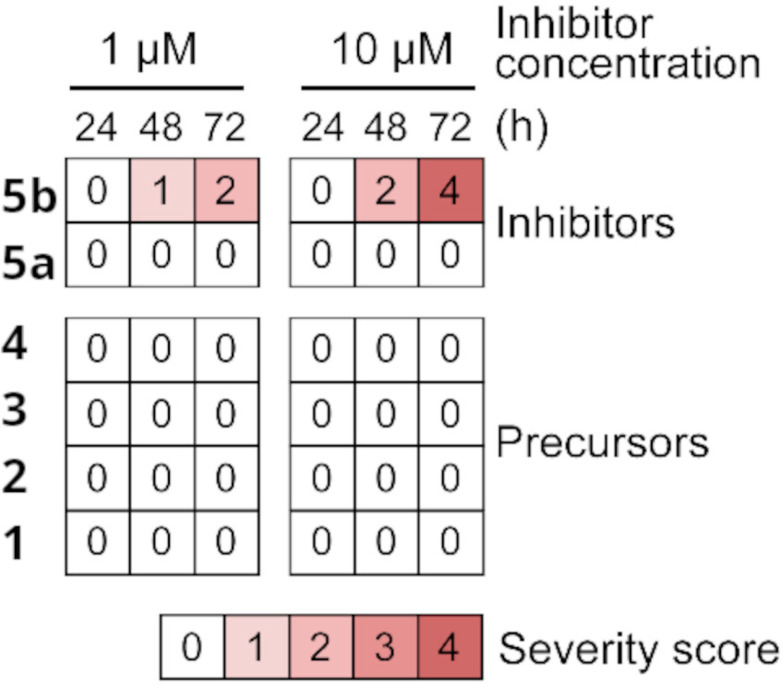
The anti-schistosomal activity of SmCB1 inhibitors and their precursors. Phenotypic changes in newly transformed schistosomula (NTS) of *S. mansoni* in the presence of 1 and 10 μM compound were recorded at three time points and converted to a severity score^24–26^ on a scale from 0 (no effect) to 4 (the most severe). The data are presented as a heat map; full phenotypic details are provided in Fig. S1A. The compounds were tested in duplicate in two independent assays (representative data are shown).

Next, the SmCB1 inhibitors 5b and 5a and their precursors were screened for bioactivity against *S. mansoni* using newly transformed schistosomula (NTS), the post-infective parasite stage that feeds on host blood and is widely used in drug screening.^47–49^ In the NTS assay, parasites were maintained in culture and exposed to 1 and 10 μM compounds for three days, and the resulting phenotypic responses were graded as a severity score on a scale of 0 (no change compared to DMSO control) to 4 (the most severe changes).^48,49^ The severity scores in Fig. 3 show that only the carborane inhibitor 5b was an effective anti-schistosomal agent, a finding consistent with its high affinity for SmCB1. 5b induced phenotypic changes at 1 μM after a 72 h incubation with NTS, and the effect was more pronounced at 10 μM, with parasite degeneration and an associated maximum severity score of 4. Notably, the efficacy of 5b was comparable to that of the cysteine protease inhibitor, K11777,^24,25^ which has previously been evaluated as an anti-schistosomal drug in *ex vivo* and *in vivo* studies.^20,24,25^ Unlike 5b, its phenyl analog, 5a, and the precursor, 4, were ineffective against NTS, as were the uncoupled carborane cage, 1 and its derivatives, 2 and 3. The data, therefore, indicate that the anti-schistosomal effect of 5b was more likely due to cathepsin inhibition than a non-specific effect of the carborane cage. The compounds were also tested for their cytotoxicity at 1 and 10 μM against four cell lines. All were essentially non-toxic (Fig. S1), demonstrating that the phenotypic changes observed in the NTS were parasite-specific.

In conclusion, the N-terminal (P3) tagging of a carborane to a peptidomimetic ligand potentiates the inhibition of SmCB1 and confers specific anti-schistosomal activity. The SmCB1 inhibitor, 5b, has been presented as an effective anti-schistosomal agent and represents, therefore, a progenitor compound for a novel class of potential drugs to treat schistosomiasis. The potency of 5b in the micromolar range remains modest compared to previously reported SmCB1 inhibitors, which have reached nanomolar inhibition potency.^24,25,50^ As a future strategy, we propose transplanting the carborane tag onto previously developed scaffolds bearing various reactive warheads, as a proof of concept for the versatility of this building block.

### Crystallographic analysis of the binding mode of 5b in the SmCB1 active site

**Fig. 4:**
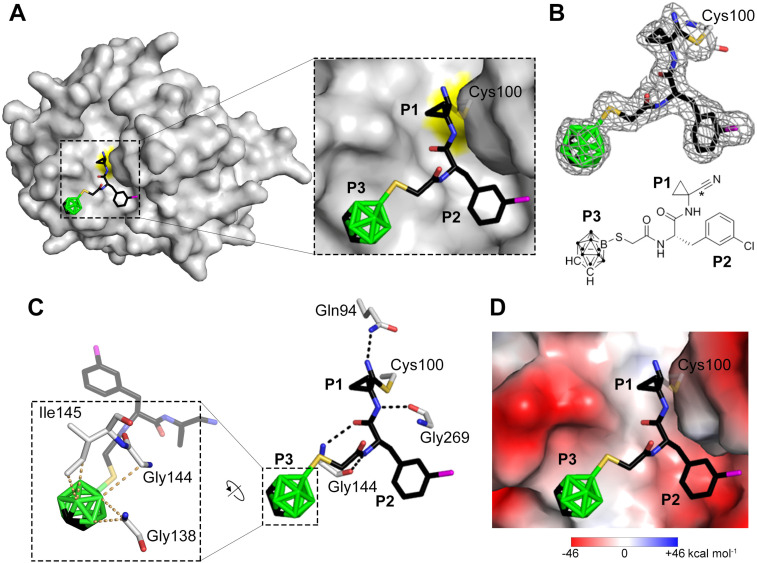
The binding mode of the carborane inhibitor 5b in the active site of SmCB1. (A) The P1–P3 positions of 5b occupy the respective enzyme binding subsites, S1–S3. The structure of SmCB1 is depicted as a surface representation with the catalytic residue, Cys100, highlighted in yellow. The inhibitor is shown in a stick representation, and the atoms are color-coded (C – black, O – red, N – blue, S – yellow, B – green, Cl – magenta). (B) The *F*_o_–*F*_c_ electron-density map of 5b is contoured at 1 σ. In the chemical structure of 5b, the carbon atom forming a covalent bond with the sulfur atom of Cys100 is marked with an asterisk. (C) The interactions of 5b in the SmCB1 active site. The right panel shows the H-bond network (black dashed lines) between SmCB1 residues (gray) and 5b (colored as in A). The zoomed view shows the carborane cage of 5b forming a network of contacts (yellow dashed lines) with SmCB1 residues; the distance cutoff was set to 110% of the sum of van der Waals radii.^53^ (D) The same view as in A, with the molecular surface of SmCB1 colored by electrostatic potential.

The crystal structure of SmCB1 in complex with the inhibitor 5b was determined at a resolution of 2.2 Å (PDB ID: 9FZV, Table S2). The binding mode of 5b in the active site is presented in Fig. 4. The P1–P3 residues of the inhibitor occupied the S1–S3 subsites^24–26^ of SmCB1. 5b formed a covalent adduct with the thiol group of the catalytic residue Cys100, which involved the carbon atom of the nitrile warhead (Fig. 1B and 4B). The thioimidate nitrogen atom was stabilized by a H-bond to the side-chain amide of Gln94, a residue forming the catalytic oxyanion hole (Fig. 4C). An analogous interaction was observed in the structures of SmCB1 and the SARS-CoV-2 main protease complexed with an azadipeptide nitrile inhibitor,^26,33^ as well as in the structures of cysteine cathepsins complexed with related dipeptidyl cyclopropyl nitrile inhibitors.^51,52^ The backbone of 5b was further stabilized by three H-bonds with Gly144 and Gly269 (Fig. 4C). The P1 position of 5b with its small cyclopropyl group provided only a few contacts with the S1 subsite. The aromatic 3-chloro-Phe substituent at the P2 position formed nonpolar interactions with Leu146 in the S2 subsite. To avoid a steric clash with this bulky substituent, Glu316 rotated out of the S2 pocket in a manner similar to that observed in SmCB1 complexed with vinyl sulfone inhibitors containing Phe at P2.^24,25^

The wide, hydrophobic S3 subsite of SmCB1 accommodated the carborane moiety of 5b (Fig. 4C). There were five possible rotational isomers of the carborane cage, which could not be unambiguously distinguished: the presented rotamer was selected based on the computational analysis of the rotational profile (Fig. S2). This cage formed a network of contacts with residues Gly138, Gly144 and Ile145 in the S3 subsite (Fig. 4C). The electron-density map for the Ile145 side-chain was not well defined, suggesting flexibility of this residue, which may be associated with the rotation of the cage. Leu139 was pushed out of the S3/S4 subsite by the bulky carborane moiety, and, thus, its position differs from that in the SmCB1 complexes with inhibitors containing sterically less demanding P3 substituents.^24–26^

### Molecular modeling

**Fig. 5:**
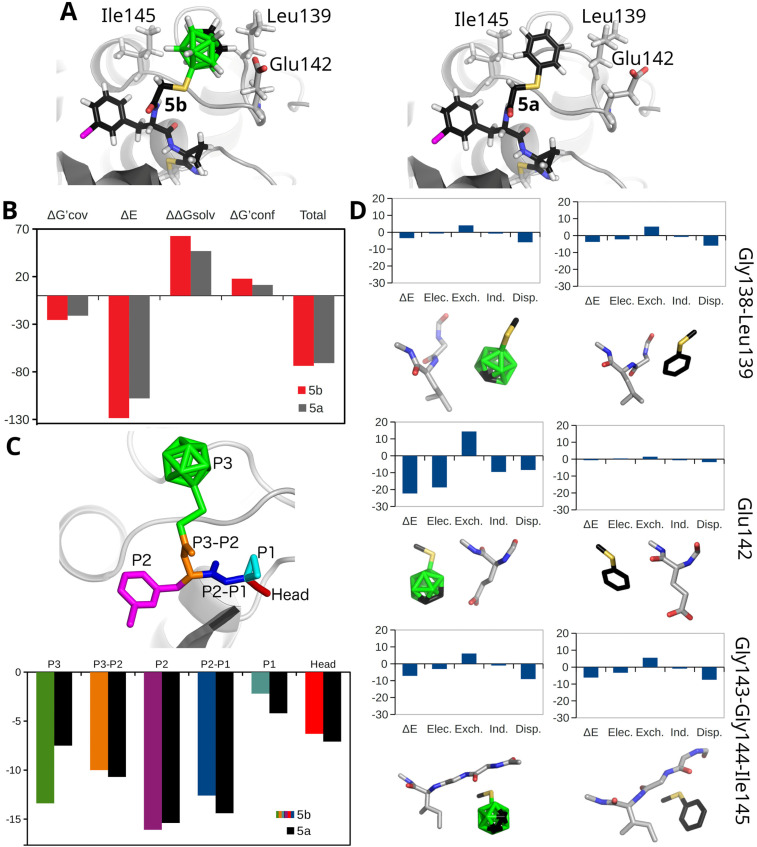
(A) The QM/MM optimized model of the 5b (left) and 5a (right) inhibitors covalently bound to SmCB1. (B) The protein–ligand score is computed as the sum of the following terms: the ‘free’-energy difference between covalent and noncovalent complexes (Δ*G*′_cov_), the interaction energy (Δ*E*), the interaction solvation free energy (ΔΔ*G*_solv_) and the change of conformational ‘free’ energy (Δ*G*′_conf_). (C) The graph displays the sums of Δ*E* and ΔΔ*G*_solv_ for individual fragments of 5b (colored by fragments) and 5a (shown in black). The fragment definitions and corresponding color coding are illustrated above the graph on inhibitor 5b. (D) The modeled noncovalent complexes of 5b (left) and 5a (right). The Δ*E* values of the P3 fragments with the surrounding amino acids have been decomposed into electrostatic (Elec.), induction (Ind.), dispersion (Disp.) and exchange (Exch.) contributions by using the SAPT0/jun-cc-pVDZ method. All energies on the *y*-axes are in kcal mol^−1^.

The crystal structure of SmCB1 in complex with compound 5b (PDB ID: 9FZV) was used as the basis for computational analysis of inhibitor binding. In the crystal structure, the S2 and S3 subsites of SmCB1, which accommodate the P2 and P3 residues of 5b, are partially solvent-exposed and may be affected by interactions with the symmetry-related molecule (Fig. S3). To address this, a computational model was constructed that excludes this crystal packing effect, representing SmCB1 in a solution-like environment. A relaxed scan along the CH/5b⋯OE1/Glu142 H-bond coordinate revealed a more favorable conformation of the SmCB1–5b complex, in which the H-bond is formed (Table S3 and Fig. 5A, S3, S4). This optimized model was used for subsequent computational analysis. We also constructed a comparative model of the SmCB1 complex with 5a, in which the carborane moiety is replaced by a phenyl ring. In this model, the formation of the H-bond between the inhibitor and Glu142 was energetically unfavorable (Table S3 and Fig. 5A, S3B).

The energetics of binding of 5b and 5a to SmCB1 were examined by means of the advanced quantum-mechanical scoring function.^54,55^ We went beyond the end-point ‘free’-energy computations and also modeled the intermediate noncovalent complexes. This approach made it possible to separate the contributions of covalent and noncovalent interactions. In contrast to the covalent complex, the residue His270 was positively charged, and the distance between the thiolate sulfur atom of Cys100 and the respective carbon atom of the inhibitor was elongated to about 3.3 Å in the noncovalent complex (Fig. S5).^24–26,55^

Compound 5b, containing the icosahedral carborane cage, exhibited a more negative computed total binding ‘free’ energy than 5a (by 2.9 kcal mol^−1^, see Fig. 5B and Table S4), consistent with the experimental *K*_i_ values for inhibition of SmCB1 (Table 1). The Δ*E* and Δ*G*′_cov_ terms for 5b were more favorable by 20.7 and 4.5 kcal mol^−1^, respectively, compared to 5a (Table S4). In contrast, the ΔΔ*G*_solv_ and Δ*G*′_conf_ terms for 5b were less favorable by 15.8 and 6.5 kcal mol^−1^, respectively. These results indicate that the improved binding affinity of 5b toward SmCB1 is primarily driven by specific molecular interactions.

To gain a deeper insight into inhibitor binding, both inhibitors were computationally fragmented into individual side-chain and main-chain segments corresponding to the P3–P1 positions, and their affinities for SmCB1 were evaluated. As shown in Fig. 5C, the sums of Δ*E* and ΔΔ*G*_solv_ for the corresponding fragments of 5b and 5a differed by less than 2 kcal mol^−1^, with the exception of the P3 fragment. The P3 fragment of 5b, which contains the carborane cage, supported ligand binding considerably more than the phenyl-containing fragment of 5a, with a difference of 5.9 kcal mol^−1^.

Furthermore, the Δ*E* values of the P3 fragments of 5b and 5a with the surrounding amino acids were evaluated by the symmetry-adapted perturbation-theory (SAPT) methodology^56^ (Table S5). The SAPT results revealed a Δ*E* improvement of −22.3 kcal mol^−1^ by the phenyl-to-carborane substitution, which is consistent with the energy difference obtained for the full enzyme (Fig. 5B). SAPT also enabled the decomposition of Δ*E* into individual energy components: the classical electrostatic interaction of two charge densities (*E*_elec_), exchange (Pauli) repulsion (*E*_exch_), electrostatic induction (the polarization of the molecular orbitals in the electric field of the interacting molecule, *E*_ind_) and dispersion (*E*_disp_) (Fig. 5D and Table S5). Notably, the largest energetic gain by the phenyl to carborane substitution occurred in the interaction with the Glu142 fragment, where Δ*E* improved from −0.7 to −22.3 kcal mol^−1^. This strong interaction corresponds to the H-bond formed between the CH vertex of the carborane cage and Glu142 in the SmCB1–5b model. The electrostatic interactions (*E*_elec_) were the dominant contributor to this binding (Fig. 5D and Table S5), underscoring the crucial role of the carborane cage's dipole moment in enhancing affinity. For the Gly143-Gly144-Ile145 fragment, the SAPT Δ*E* improved from −6.2 to −7.2 kcal mol^−1^ (Table S5) upon the phenyl-to-carborane substitution. The interaction of the carborane cage with the Gly138-Leu139 fragment was only slightly weaker than that of the phenyl (−3.5 *vs.* −3.8 kcal mol^−1^, Table S5), suggesting a comparable level of binding in that region. The interaction of the cage with both fragments was primarily stabilized by dispersion, which constituted 69% and 79% of the sum of all of the attractive energy terms in the SAPT decomposition for the Gly143-Gly144-Ile145 and Gly138-Leu139 fragments, respectively (Fig. 5D and Table S5). These results indicate that the carborane cage establishes a network of nonpolar BH⋯HC interactions for both the Gly143-Gly144-Ile145 and Gly138-Leu139 fragments. This finding is consistent with the crystallographic analysis of carborane binding in the SmCB1 active site (Fig. 4). Although each BH⋯HC interaction is relatively weak, their cumulative effect significantly contributes to binding affinity.

In addition to the B(9)–S linkage between the carborane cage and the peptidomimetic scaffold utilized in 5b, a structural model of SmCB1 complexed with a hypothetical inhibitor was prepared in which the carborane cage is connected *via* the C(1) atom. The noncovalent interactions of the 1-CH_3_S-*closo*-1,2-C_2_B_10_H_11_ fragment were examined at the SAPT level. The obtained results revealed weaker interactions of the carborane cage with the Gly138-Leu139 and Glu142 fragments compared to the B(9)-linked variant, with interaction energy decreasing from −3.5 to −2.6 and from −22.3 to −3.7 kcal mol^−1^ respectively, (see Tables S5, S6 and Fig. 5D, S6). On the other hand, the interaction with the Gly143-Gly144-Ile145 fragment was slightly enhanced, with an interaction energy improving from −7.2 to −7.6 kcal mol^−1^ (Tables S5 and S6). Overall, the total SAPT interaction energy of the C(1)-linked carborane cage was −14.0 kcal mol^−1^, which is less favorable than that of the B(9)-linked configuration (−33.0 kcal mol^−1^). Nevertheless, both orientations of the carborane cage provided more favorable interaction energies than the phenyl analog (−10.7 kcal mol^−1^; Tables S5 and S6).

**Fig. 6:**
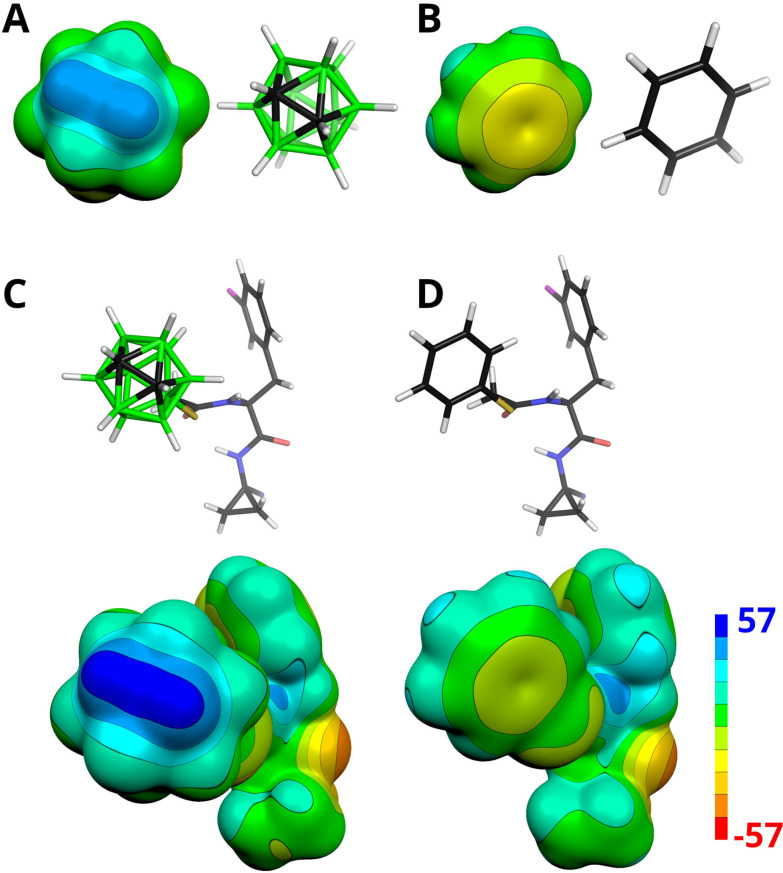
The electrostatic potential (ESP) molecular surface of (A) *closo*-1,2-C_2_B_10_H_12_, (B) benzene, (C) 5b, and (D) 5a computed at the HF/cc-pVDZ level. The ESP color range is in kcal mol^−1^. Note that the highly positive (blue) ESP of the CH vertices of **1** indicates their acidity, which predisposes them to interact favorably with negatively charged sites.

The results highlight the critical importance of the presence of the carborane cage's dipole moment in mediating the binding of 5b. Notably, the computed electrostatic potential (ESP) molecular surfaces revealed that the CH vertices of the carborane cage in 5b are more positive compared to those in 1 with magnitudes of the ESP molecular surface (*V*_S,max_) values of 51.7 and 42.3 kcal mol^−1^, respectively (Fig. 6). This observation aligns with previous findings that the functionalization of the BH groups antipodally coupled to the CH vertices in 1 increased the dipole moment. For example, the 9-phenyl *ortho*-carborane has a dipole moment value of 5.0 D, approximately 0.5 D more than that of unsubstituted *ortho*-carborane (1).^13^

Finally, we compared the electrostatic character of the S3 binding subsite in SmCB1 with those in other cysteine proteases inhibited by 5b/5a (Table S1). Among the proteases analyzed, cathepsin S was the only one which had a positive side-chain (Lys) near the position of the CH vertices of the carborane when its structure was aligned with the SmCB1–5b complex. The remaining cathepsins had negatively charged side-chains of either Glu or Asp at this location. This finding correlates with their enhanced inhibition by 5b compared to 5a, a trend that is reversed only for cathepsin S, which is more sensitive to inhibition by 5a (Table S1). These results highlight the importance of H-bonding for the binding of the carborane cage of 5b.

## Conclusions

We demonstrate that the icosahedral *ortho*-carborane tag improves the functional properties of a peptidomimetic ligand compared to its 2D phenyl analog, a conventional substituent in drug design. This was specifically shown by both the more potent inhibition of the SmCB1 protease target and enhanced bioactivity against the *S. mansoni* parasite. Quantum mechanical computations based on the crystal structure of the inhibitor–protease complex revealed that, in addition to nonpolar interactions, the carborane tag provided a favorable H-bond between the CH vertex of the carborane cage and the negatively charged Glu142 side-chain in the enzyme. This interaction was driven by the substantial dipole moment of the carborane cage. The carborane tag also improved inhibition of other parasite- and human-derived cathepsin proteases, except for cathepsin S, which contains the positively charged subsite. Overall, the carborane substituent represents a valuable pharmacophore with the potential to enhance ligand affinity for protein binding sites with specific spatial and electrostatic requirements.

## Methods

### Ethical statement

*Schistosoma mansoni* (NMRI strain) was maintained in the CDIPD by cycling between *Biomphalaria glabrata* snails and Golden Syrian hamsters.^20,57^ Vertebrate animal use is supported under a protocol approved by UC San Diego's Institutional Animal Care and Use Committee. The protocol complies with United States federal regulations regarding the care and use of laboratory animals: Public Law 99-158, the Health Research Extension Act, and Public Law 99-198, the Animal Welfare Act, which is regulated by USDA, APHIS, CFR, Title 9, Parts 1, 2, and 3.

### General enzymatic methods

Enzymatic activity was monitored either fluorometrically with peptide–AMC substrates, which release 7-amino-4-methylcoumarin (AMC) as a fluorescent product, or spectrophotometrically with peptide–pNA substrates, which liberate *p*-nitroaniline (pNA). Reaction rates were obtained from the slopes of the linear progress curves and expressed as relative fluorescence units per second (RFU s^−1^) or as absorbance units per second (AU s^−1^). *K*_i_ values for inhibitors were calculated by non-linear regression with the equation *v*_i_/*v*_0_ = 1/(1 + [I]/*K*_i_ (1 + [S]/*K*_m_)) using GraphPad Prism software, where *v*_i_ is the product formation rate in the presence of given concentration of inhibitor, *v*_0_ the product formation rate in the absence of inhibitor, [I] the inhibitor concentration, [S] the substrate concentration, *K*_m_ the Michaelis constant, and *K*_i_ the inhibition constant. The standard errors of measurements refer to the non-linear regression analysis.

### SmCB1 inhibition assay

A non-glycosylated mutant of the SmCB1 zymogen was expressed in the yeast *Pichia pastoris*, activated by *S. mansoni* legumain and purified as described previously.^25,43,58^ Kinetic measurements were performed in triplicate in 96-well microplates (100 μL assay volume) at 37 °C. SmCB1 (40 pM) was added to a mixture of the fluorogenic substrate Cbz-Phe-Arg-AMC (40 μM = 1.6 × *K*_m_) and an inhibitor (0–200 μM) in 100 mM sodium acetate, pH 5.5, 2.5 mM DTT, 0.01% Brij 35. Substrate hydrolysis was measured fluorometrically in an Infinite M1000 microplate reader (Tecan) at excitation and emission wavelengths of 360 and 465 nm, respectively. Inhibition assays for human cathepsins and cruzain are described in Supplemental Experimental Procedures.

### Preparation of the SmCB1–5b complex

The SmCB1 zymogen (0.6 mg mL^−1^) was activated by *S. mansoni* legumain and simultaneously inhibited by a 5-fold molar excess of 5b in 50 mM sodium acetate, pH 5.0, 20 mM cysteine, 1 mM EDTA under argon atmosphere as described previously.^43^ The enzyme inhibition was monitored using a kinetic assay with the fluorogenic substrate Cbz-Phe-Arg-AMC.^43^ The complex was chromatographed on an FPLC Mono S column (Cytiva) in 25 mM MES, pH 6.3, 2.5 mM DTT, 1 mM EDTA, 3 μM 5b, then buffer-exchanged into 10 mM sodium acetate, pH 5.5, 2.5 mM DTT, 180 μM 5b and concentrated to a final concentration of 5 mg mL^−1^ using Amicon Ultracel-10k centrifugal units (Millipore).

### Crystallization of the SmCB1–5b complex, data collection and structure determination

Crystals of the SmCB1 complex with 5b were obtained by vapor diffusion in a hanging drop. Diffraction data were collected using synchrotron radiation. The structure was determined by molecular replacement using the structure of SmCB1 (PDB ID: 4I07)^59^ as the search model and refined using data to a resolution of 2.2 Å. For details, see Supplemental Experimental Procedures. Data collection and refinement statistics are given in Table S2. Atomic coordinates and structure factors were deposited in the Protein Data Bank with accession code 9FZV.

### Phenotypic assay with *Schistosoma mansoni* Schistosomula

Newly transformed schistosomula (NTS) of *S. mansoni* were prepared by mechanically transforming infective larvae (cercariae) as described previously.^47,57^ NTS (200–300 parasites) were incubated in flat-bottomed 96-well plates in 200 μL of Basch medium 169^60^ containing 5% FBS (ThermoFisher Scientific), 100 U mL^−1^ penicillin and 100 μg mL^−1^ streptomycin (ThermoFisher Scientific), at 5% CO_2_ and 37 °C.^61,62^ Inhibitors were added at final concentration of 1 or 10 μM,^43,47^ and changes in phenotype were observed every 24 h for up to 72 h. A constrained nomenclature of ‘descriptors’^49,57^ is used to record the multiple and dynamic changes in movement, shape and translucence that the schistosome parasite is capable of (Fig. S1). These descriptors are then converted into an ordinal ‘severity score’ system from 0 (no effect) to 4 (maximum effect), which allows for the relative comparison of compound effects, as described previously.^63^

### Molecular modeling

The crystal structure of SmCB1 in complex with 5b (PDB ID: 9FZV) was used for the modeling process. Hydrogen atoms were added to the protein structure by the Reduce and Leap programs in AMBER 14.^64^ Aspartates, glutamates, lysines, arginines, and histidines were charged, except for the neutral His176, His270 (in the covalent complex), Glu179, and Glu242. 5a was built manually from 5b, and the H atoms of the 5b were added manually as well. The ff19SB force field^65^ was used for the protein. A general AMBER force field (GAFF) was combined with the boron parameters from the universal force field (UFF) for the ligand.^66^ Added atoms were relaxed by annealing from 900 K to 0 K at the molecular mechanics (MM) level in AMBER 20. The annealing protocol employed a Berendsen thermostat over 4 ps with a 1 fs time step.

The studied complexes were optimized using the QM/MM hybrid approach. The QM part comprised the inhibitor, Glu142, and all residues within 2.5 Å of the 5b inhibitor. The QM part was treated at the DFT-D3/BLYP/DZVP-DFT/COSMO level for energy calculations (the DZVP-DFT basis set is available through the basis set exchange repository, https://www.basissetexchange.org, as dgauss-DZVP).^67^ The remainder of the system was treated at the MM level with the IGB7 implicit solvent model by AMBER.^64^ The coupling between QM and MM was done by Cuby4,^68^ which calls Turbomole 7.3^69^ and AMBER^64^ for QM and MM, respectively. Residues further than 6 Å of 5b were frozen during the optimization. Since B–H and C–H vertices are difficult to distinguish by X-ray crystallography^70^ all the rotamers of the carborane cage were modeled and the most stable one was used further. To examine the interaction between the P3 moiety of the 5b/5a inhibitors and the Glu142 side chain, a relaxed scan was performed along the CH⋯OE1/Glu142 H-bond coordinate. This scan applied a harmonic restraint on the CH⋯O distance in increments of 0.3 Å while allowing the remaining degrees of freedom to relax. The scan was necessary due to the loss of symmetry-related hydrogen bonding partners in the crystal environment.

The noncovalent complexes of 5b and 5a with SmCB1 were generated by breaking the covalent bond between the inhibitor and the SG atom of Cys100 and transferring the proton from the inhibitor to His270. This process was achieved by performing a relaxed scan along the proton transfer trajectory.^55^ The resulting complexes were fully reoptimized without restraints and evaluated using the QM-based scoring function.^55^ The energy difference between the covalent and noncovalent complexes corresponds to the Δ*G*′_cov_ term of the total score, which also includes the gas-phase interaction energy (Δ*E*), the interaction desolvation free energy (ΔΔ*G*_solv_), and the change in the conformational ‘free’ energy of the ligand (Δ*G*′_conf_).

The Δ*E* values of the selected motif were decomposed using the symmetry-adapted perturbation-theory (SAPT) method.^56^ The simplest truncation of the SAPT (SAPT0) decomposition was performed with the recommended jun-cc-pVDZ basis set using the PSI4 program.^71^ The molecular ESP surfaces were computed on the 0.001 a.u. electron density isosurface at the HF/cc-pVDZ level using Gaussian09^72^ and visualized with Molekel4.3.^73,74^

## Author contributions

J. H., M. G., D. H., M. M., and J. F. conceived and designed the project. J. H. synthesized the carboranethiol and conjugated it to the ligand scaffold. C. L., L. C., C. B., and F. R. R. prepared and functionalized the ligand scaffold. A. J. and M. H. prepared SmCB1, P. S. crystalized the SmCB1 complex, and A. J. and J. B. solved its crystal structure. C. L., P. S., M. H., F. R. R., and C. M. performed biochemical and enzymological experiments. H. M.-K. conducted cytoxicity screening, A. L., M. C., N. E.-S, and C. R. C. evaluated the anti-schistosomal activity. D. H. and J. F. performed molecular modeling and quantum mechanical studies. J. H., A. J., M. H., M. G., D. H., M. M., and J. F. drafted the manuscript with contributions from all co-authors. All authors analyzed the data, reviewed, and approved the final manuscript.

## Conflicts of interest

The authors declare no conflict of interest.

## Supplementary Material

QI-013-D5QI01546D-s001

## Data Availability

The data supporting this article have been included as part of the supplementary information (SI). Supplementary information: experimental details of the compound synthesis, protein crystallization, data collection, refinement, and analysis, inhibition assays, supplementary tables S1–S6, figures S1–S26, and references. See DOI: https://doi.org/10.1039/d5qi01546d.
