# Supplementary material for: Fat or flat? The impact of dipole moment vectors on non-covalent interactions between aromatic tags and macromolecules
Source: Inorg Chem Front. 2025 Oct 21;13(2):364–76. doi: 10.1039/d5qi01546d (PMC12538275; doi:10.1039/d5qi01546d)
Supplement: QI-013-D5QI01546D-s001 [file QI-013-D5QI01546D-s001.pdf]

Supplementary Information (SI) for Inorganic Chemistry Frontiers.
This journal is © the Partner Organisations 2025

Supplementary Information for

Fat or Flat? The Impact of Dipole Moment Vectors on Non-Covalent Interactions Between Aromatic Tags and Macromolecules

Josef Holub,[‡a] Adéla Jílková,[‡b] Carina Lemke,[‡c] Lorenzo Cianni,[‡d] Petra Spiwoková,[b] Martin Horn,[b] Christian Breuer,[c] Adrian Leontovyč,[b] Jiří Brynda,[b] Helena Mertlíková-Kaiserová,[b] Marta Chanová,[e] Fernanda dos Reis Rocho,[d] Carlos Montanari,[d] Nelly El-Sakkary,[f] Conor R. Caffrey,[f] Michael Gütschow,*[c] Drahomír Hnyk,*[a] Michael Mareš,*[b] and Jindřich Fanfrlík*[b]

[a] *Institute of Inorganic Chemistry of the Czech Academy of Sciences, Husinec-Řež 250 68, Czech Republic. E-mail: hnyk@iic.cas.cz (D.H.)*

[b] *Institute of Organic Chemistry and Biochemistry of the Czech Academy of Sciences, Flemingovo nám. 2, 16610 Prague 6, Czech Republic. E-mail addresses: mares@uochb.cas.cz (M.M.) and fanfrlik@uochb.cas.cz (J.F.)*

[c] *Pharmaceutical Institute, Pharmaceutical & Medicinal Chemistry, University of Bonn, An der Immenburg 4, 53121 Bonn, Germany. E-mail: guetschow@uni-bonn.de (M.G.)*

[d] *Medicinal and Biological Chemistry Group, São Carlos Institute of Chemistry, University of São Paulo, Avenue Trabalhador Sancarlense, 400, 13566-590, São Carlos/SP, Brazil.*

[e] *Institute of Immunology and Microbiology, First Faculty of Medicine, Charles University and General University Hospital in Prague, Viničná 7, 12844 Prague 2, Czech Republic.*

[f] *Center for Discovery and Innovation in Parasitic Diseases, Skaggs School of Pharmacy and Pharmaceutical Sciences, University of California San Diego, 9255 Pharmacy Lane, MC0657, La Jolla, CA 92093, United States.*

[‡]These authors contributed equally.

Table of Content

Experimental Procedures……… S3

General Synthetic Methods and Materials……… S3

Syntheses and Characterizations……… S4

Protein Crystallization and Data Collection……… S7

Structure Determination, Refinement, and Analysis……… S7

Enzymatic Assay Materials……… S8

Cathepsin B Inhibition Assay……… S8

Cruzain Inhibition Assay……… S8

Cathepsin K Inhibition Assay……… S8

Cathepsin L Inhibition Assay……… S9

Cathepsin S Inhibition Assay……… S9

Supporting Tables……… S10

Table S1. Inhibitory affinity against selected cathepsin L-type cysteine proteases… S10

Table S2. X-ray data collection and refinement statistics……… S11

Table S3. QM/MM relative 'free' energies score……… S12

Tables S4. QM/MM-based protein-ligand score……… S12

Table S5, S6. Interaction energy decomposition……… S12

Supporting Figures……… S13

Fig. S1. Anti-schistosomal activity, and cytotoxicity……… S13

Fig. S2. Relative 'free' energy plotted against the C7-S1-C17-C16 torsion……… S14

Fig. S3. Relaxed scan along the CH/**5b**—OE1/Glu142 H-bond……… S15

Fig. S4. Glu142 in the SmCB1 active site……… S16

Fig. S5. Structural details of the modeled noncovalent SmCB1–**5b** complex……… S17

Fig. S6. Modeled C(1)-linked carborane complex……… S18

Fig. S7. ^{1}H NMR spectrum of compound **2**……… S19

Fig. S8. ^{13}C NMR spectrum of compound **2**……… S20

Fig. S9. ^{11}B NMR spectrum of compound **2**……… S21

Fig. S10. ^{1}H NMR spectrum of compound **3**……… S22

Fig. S11. ^{13}C NMR spectrum of compound **3**……… S23

Fig. S12. ^{11}B NMR spectrum of compound **3**……… S24

Fig. S13. ^{1}H NMR spectrum of compound **4**……… S25

Fig. S14. ^{13}C NMR spectrum of compound **4**……… S26

Fig. S15. HPLC–MS chromatogram of compound **4**……… S27

Fig. S16. HPLC-HRMS spectrum of compound **4**……… S28

Fig. S17. ^{11}B NMR spectrum of compound **5b**……… S29

Fig. S18. ^{1}H NMR spectrum of compound **5b**……… S30

Fig. S19. ^{13}C NMR spectrum of compound **5b**…………….. S31
Fig. S20. HPLC chromatograms of compound **5b**.. S32
Fig. S21. HPLC-HRMS spectrum of compound **5b**... S33
Fig. S22. ^{1}H NMR spectrum of compound**5a**.. S34
Fig. S23. ^{13}C NMR spectrum of compound **5a**... S35
Fig. S24. HPLC–MS chromatogram of compound **5a**.. S36
Fig. S25. HPLC-HRMS spectrum of compound **5a**;.. S37
Fig. S26. Vertex numbering of *closo*-1,2-$C_2B_{10}H_{12}$ (**1**)………………….……….. S38
References…………………………………………………………………….…...… S39

Experimental Procedures

General Synthetic Methods and Materials

The syntheses were performed at the Institute of Inorganic Chemistry in the Czech Republic and at the University of Bonn in Germany. Thin-layer chromatography (TLC) was carried out on Merck aluminum silica gel plates with 60 F_{254} indicator. Detection was performed with UV light at 254 nm or 366 nm. Preparative column chromatography was performed using Merck silica gel 60 (63-200 mesh). Melting points were determined on a Büchi 510 oil bath apparatus and were uncorrected.

^{1}H NMR, ^{13}C NMR, and ^{11}B NMR spectra for precursors **2** and **3** were obtained using a Varian machine at 400 MHz in $CDCl_3$ in the Institute of Inorganic Chemistry, Czech Republic and the ^{11}B NMR chemical shifts were related to external $BF_3 \cdot Et_2O$. Numbering scheme related to all ^{11}B NMR spectra is given in Fig. S26. ^{1}H NMR (600 MHz or 500 MHz) and ^{13}C NMR (150 MHz or 125 MHz) spectra for compounds **4**, **5a** and **5b** (and ^{11}B NMR spectra when applicable) were recorded on a Bruker Avance III-600 MHz or Bruker Avance DRX-500 MHz instrument at 30 °C using DMSO-d_6 or at 25 °C using $CDCl_3$ as solvent at the University of Bonn. Chemical shifts reported from the both laboratories are in ppm relative to the remaining protons of the deuterated solvent used as an internal standard (DMSO-d_6: 2.49 / 39.7 ppm; $CDCl_3$: 7.24 / 77.0 ppm) and multiplicities are given as s (singlet), d (doublet), t (triplet), q (quartet), quint (quintet) and m (multiplet).

HPLC (DAD) chromatograms and MS (ESI) spectra were recorded on an API 2000 mass spectrometer (AB Sciex, Darmstadt, Germany) coupled with an Agilent HP1100 HPLC system using an EC50/2 Nucleodur C18 Gravity 3 µm column (Macherey-Nagel, Düren, Germany) at the University of Bonn. The purity of the tested compounds was determined monitoring the UV absorption from 220 nm to 400 nm on the described system using a procedure as follows: samples were dissolved (1 mg mL^{-1}) in MeOH containing 2 mM NH_4+ CH_3COO^-. Then, 8 µL of the sample solution was injected into the column at 25 °C. The mobile phase was a mixture of H_2O containing 2 mM NH_4^+ CH_3COO^- (A) and MeOH containing 2 mM NH_4^+ CH_3COO^- (B). Elution was performed following a gradient of A/B (90:10) to (0:100) in 10 min, then (0:100) to 20 min at a flow rate of 300 µL min^{-1}. HRMS (ESI) spectra were recorded on a microTOF-Q mass spectrometer (Bruker, Köln, Germany) coupled with an HPLC Dionex Ultimate 3000 (Thermo Scientific, Braunschweig, Germany) using an EC50/2 Nucleodur C18 Gravity 3 µm column (Macherey-Nagel, Düren, Germany). Samples were dissolved (0.6 mg

mL-1) in MeCN at the University of Bonn. Then, 1 μL of the sample solution was injected into the column at 25 °C. The mobile phase was a mixture of H_2O containing 2 mM NH_4^+ CH_3COO^- (A) and MeCN (B). Elution was performed following a gradient of A/B (90:10) for 1 min, then (90:10) to (0:100) in 9 min and (0:100) to 20 min at a flow rate of 300 μL min^{-1}.

Reagents were purchased from Abcr and solvents as well as $AlCl_3$ from Sigma-Aldrich, and were used without further purification. Carborane **1** was purchased from Katchem, Ltd. in the declared purity of 99%, sulfur was purchased from Lach-Ner.

Syntheses and Characterizations

*9-SH-closo-1,2-$C_2B_{10}H_{11}$ (**2**).* Precursor **2** was prepared from *closo*-1,2-$C_2B_{10}H_{12}$ (**1**; 1.44 g, 0.01 mol), $AlCl_3$ (1.60 g, 0.012 mol) and elemental sulfur (0.80 g, 0.025 mol), which were mixed and melted together in an autoclave at 120 °C for 5 h.[1] Precursor **2** (950 mg, 540 mmol, 54%) was isolated from the reaction mixture. ^{11}B NMR (400 MHz, $CDCl_3$, ^{1}H-decoupled) δ -16.4 (2d, **B3,6**), -15.3 (2d, **B7,11**), -14.3 (2d, **B4,5**), -8.9 (2d, **B8,10**), -2.3 (1d, **B12**), 3.9 (1s, **B9**); ^{1}H NMR (400 MHz, $CDCl_3$, ^{11}B-decoupled) δ 0.42 – 2.88 (m, 10H, B-H, SH), 3.43 (s, 1H, **C2**-H), 3.59 (s, 1H, **C1**-H); ^{13}C NMR (100 MHz, $CDCl_3$, ^{11}B-decoupled) δ 47.43 (**C1**), 53.79 (**C2**).

*9-SCH_2CO_2tBu-closo-1,2-$C_2B_{10}H_{11}$ (**3**).* Precursor **2** (1.76 g, 10.0 mmol) was dissolved in dry diethylether (50 mL). To the solution, 1.0 g of NaH (60% dispersion in mineral oil) was added upon hydrogen evolution. The reaction mixture was stirred for 2 h, then the excess of NaH was filtered off. *tert*-Butyl bromacetate (2.5 g, 12.8 mmol) was added and it was stirred for 24 h. The reaction mixture was evaporated to dryness on silica gel and purified by column chromatography using benzene as an eluent to obtain **3** (2.46 g, 8.5 mmol, 85%) as a white powder. ^{11}B NMR (400 MHz, $CDCl_3$), ^{1}H-decoupled) δ -16.2 (2d, **B3,6**), -15.3 (2d, **B7,11**), -14.8 (2d,**B4,5**), -9.7 (2d, **B8,10**), -3.5 (1d, **B12**, 5.7 (1s, **B9**); ^{1}H NMR (400 MHz, $CDCl_3$, ^{11}B-decoupled) δ 1.43 (s, 9H, $CO(CH_3)_3$), 2.13 – 2.45 (m, 9H, B_9H_9), 3.14 – 3.22 (m, 2H, CH_2), 3.56 – 3.69 (m, 2H, **C2**-H, **C1**-H); ^{13}C NMR (100 MHz, $CDCl_3$, ^{11}B-decoupled) δ 28.33 (($CO_2C(\mathit{C}H_3)_3$), 35.92 (CH_2), 48.52 (**C1**), 53.70 (**C2**) 81.67 ($CO_2\mathit{C}(CH_3)_3$), 170.25 ($\mathit{C}O_2C(CH_3)_3$).

*(S)-tert-Butyl 3-(3-chlorophenyl)-1-(1-cyanocyclopropylamino)-1-oxopropan-2-yl-carbamate (**4**).* The salt aminocyclopropane-carbonitrile hydrochloride (356 mg, 3.0 mmol) was added to a solution of (*S*)-Boc-3-chlorophenylalanine (989 mg, 3.3 mmol), HATU (1.52 g, 4.0 mmol) and *N,N*-disopropylethylamine (776 mg, 1.02 mL, 6.0 mmol) in DMF (20 mL) under argon atmosphere. The resulting solution was stirred at room temperature for 20 h. The reaction mixture was diluted with ethyl acetate (150 mL) and washed with a saturated $NaHCO_3$ solution (3 × 40 mL) and brine (3 × 40 mL). The organic phase was dried over Na_2SO_4 and evaporated to give a crude residue that was purified by flash column chromatography (petroleum ether: EtOAc, 3:7) to obtain a white solid (200 mg, 0.55 mmol, yield 83%); mp 146-147 °C; lit. mp 173-177 °C.[2] ^{1}H NMR (500 MHz, $CDCl_3$) δ 1.05 – 1.13 (m, 2H, CH_2CH_2), 1.41 (s, 9H, $C(C\mathit{H}_3)_3$), 1.44 – 1.52 (m, 2H, CH_2CH_2), 2.96 – 3.04 (m,

2H, NHCHC*H*$_2$), 4.25 (br s, 1H, NHC*H*CH$_2$), 5.22 (br s, 1H, CON*H*CH), 7.03 – 7.08 (m, 2H, H_{arom}), 7.16 (br s, 1H, H_{arom}), 7.22 (br s, 1H, H_{arom}), 7.23 (br s, 1H, CON*H*C(CH$_2$)$_2$); ^{13}C NMR (151 MHz, $CDCl_3$) δ 16.56 (*C*H$_2$CH$_2$), 16.68 (CH$_2$*C*H$_2$), 20.14 (*C*(CH$_2$)$_2$), 28.18 (CO$_2$C(*C*H$_3$)$_3$), 37.87 (NHCH*C*H$_2$), 55.28 (1C, NH*C*HCO), 80.55 (CO$_2$*C*(CH$_3$)$_3$), 119.48 (CN), 127.25 (C_{arom}), 127.49 (C_{arom}), 129.29 (C_{arom}), 134.32 (C_{arom}), 138.23 (C_{arom}), 155.61 (NH*C*O$_2$C(CH$_3$)$_3$, 172.02 (CH*C*ONH), LC-MS (ESI) (60% H_2O to 100% MeOH in 10 min, then 100% MeOH over 10 min, DAD 220-450 nm), 100% purity, *m/z* = 364.3 ([M+H]$^+$), 381.1 ([M+NH$_4$]$^+$). HRMS (MicroTof QIII), calcd for $C_{18}H_{22}ClN_3O_3$ 363.84, found 364.1425 (100%), 366.1399 (32%).

(S)-3-(3-Chlorophenyl)-N-(1-cyanocyclopropyl)-2-(2-(closo-$C_2B_{10}H_{11}$-9-thio)acetamido)-propanamide (***5b***). Compound **4** (100 mg, 0.27 mmol) was dissolved in formic acid (3 mL) and stirred at room temperature overnight. The reaction mixture was evaporated under vacuum to get a yellowish oil. It was treated with 1M NaOH until pH 9. The product was extracted with ethyl acetate (4 × 25 mL) and then washed with brine (2 × 25 mL). The organic phase was dried over Na_2SO_4 and evaporated to obtain a colorless oil, which was used for the next step without any further purification (66 mg, 0.25 mmol, 92%). Compound **3** (58 mg, 0.20 mmol) was dissolved in dry CH_2Cl_2 (10 mL) and treated with trifluoroacetic acid (10 mL). After stirring for 2 h, the solvent was removed under reduced pressure and co-evaporated with CH_2Cl_2 (3 × 20 mL). Under argon atmosphere, HATU (103 mg, 0.27 mmol) and *N*,*N*-disopropylethylamine (70 mg, 92 µL, 0.54 mmol) in DMF (3.0 mL) were added to the residue, followed by the addition of the deprotected amine (45 mg, 0.17 mmol). The resulting solution was stirred at room temperature for 20 h. The reaction mixture was diluted with EtOAc (25 mL) and petroleum ether (25 mL) and washed with saturated $NaHCO_3$ solution (3 × 20 mL) and brine (3 × 20 mL). The organic phase was dried over Na_2SO_4 and evaporated to give a crude residue that was purified by flash column chromatography (petroleum ether: EtOAc, 3:7) to obtain a colorless oil (38 mg, 0.079 mmol, 46%). ^{11}B NMR (128 MHz, $CDCl_3$), ^{1}H-decoupled) δ -15.0 (m, **B3,6,4,5,7,11**), -2.9 (2d, **B8,10**), -1.7 (1d, **B12)**, 6.1 (1s, **B9**), ^{1}H NMR (500 MHz, $CDCl_3$) δ 1.05 – 1.18 (m, 2H, CH$_2$CH$_2$), 1.44 – 1.57 (m, 2H, CH$_2$CH$_2$), 1.52 – 3.09 (m, br, 9H, B$_9$*H*$_9$), 3.09 – 3.14 (m, 2H, SCH$_2$), 3.17 – 3.21 (m, 2H, NHCHC*H*$_2$), 3.59, 3.84 (each s, each 1H, **C2**-H, **C1**-H), 4.67 – 4.73 (m, 1H, NHC*H*CH$_2$), 7.11 – 7.17 (m, 1H, H_{arom}), 7.21 – 7.33 (m, 3H, H_{arom}), 7.63 (m, 2H, NH). ^{13}C NMR (151 MHz, $CDCl_3$) δ 16.36 (*C*H$_2$CH$_2$), 17.17 (CH$_2$*C*H$_2$), 20.16 (*C*(CH$_2$)$_2$), 35.99 (SCH$_2$), 36.30 (NHCH*C*H$_2$), 48.63 (1C, **C1**), 53.68 (1C, **C2**), 54.06 (NH*C*HCO), 119.69 (CN), 127.41 (*C*$_{arom}$), 127.68 (C_{arom}), 129.48 (C_{arom}), 130.09 (C_{arom}), 134.51 (C_{arom}), 138.03 (C_{arom}), 170.55 (CONH), 170.90 (CONH). LC-MS (ESI) (9% H_2O to 100% MeOH in 10 min, then 100% MeOH over 10 min, DAD 220 – 450 nm), 98% purity, *m/z* = 481.2 ([M+H]$^+$), 498.4 ([M+NH$_4$]$^+$). HRMS (MicroTof QIII), calcd for $C_{17}H_{26}B_{10}ClN_3O_2S$ 481.24, found 481.2486 (100%).

(S)-3-(3-Chlorophenyl)-N-(1-cyanocyclopropyl)-2-(2-(phenylthio)acetamido)propanamide (***5a***). Compound **4** (270 mg, 0.74 mmol) was dissolved in formic acid (6 mL) and stirred at room temperature overnight. The reaction mixture was evaporated under vacuum to get a yellowish oil. It was treated with 1M NaOH until pH 9. The product was extracted with ethyl acetate (4 × 50 mL) and then washed with brine (2 × 50 mL). The organic

phase was dried over Na_2SO_4 and evaporated to obtain a colorless oil, which was used for the next step without any further purification (189 mg, 0.72 mmol, 97%). *tert*-Butyl 2-(phenylthio)acetate (146 mg, 0.65 mmol) was dissolved in dry CH_2Cl_2 (10 mL) and treated with trifluoroacetic acid (10 mL). After stirring for 2 h, the solvent was removed under reduced pressure and co-evaporated with CH_2Cl_2 (3 × 20 mL). Under argon atmosphere, a solution of the deprotected amine (189 mg, 0.72 mmol), HATU (321 mg, 0.84 mmol) and *N*,*N*-disopropylethylamine (218 mg, 294 µL, 1.69 mmol) in DMF (5 mL) was added. The mixture was stirred at room temperature for 20 h. It was diluted with ethyl acetate (50 mL) and washed with saturated $NaHCO_3$ solution (3 × 20 mL) and brine (3 × 20 mL). The organic phase was dried over Na_2SO_4 and evaporated to give a crude residue that was purified by flash column chromatography (petroleum ether: EtOAc, 3:7) to obtain a yellowish solid (80 mg, 0.19 mmol, 30%). mp 186-187 °C. ^{1}H NMR (500 MHz, DMSO-d_6) δ 0.93 – 1.00 (m, 2H, CH_2CH_2), 1.42 – 1.47 (m, 2H, CH_2CH_2), 2.79 (dd, ^2J = 13.5 Hz, ^3J = 8.5 Hz, 1H, -HCHCH_2), 2.89 (dd, ^2J = 13.5 Hz, ^3J = 6.0 Hz, 1H, NHCHCH_2), 3.64 (s, 2H, SCH_2), 4.37 – 4.42 (m, 1H, NHC*H*CH_2), 7.09 – 7.17 (m, 2H, H_{arom}), 7.22 – 7.27 (m, 7H, H_{arom}), 8.45 (d, ^3J = 8.0 Hz, 1H, CON*H*CH), 8.93 (s, 1H, CON*H*C$(CH_2)_2$). ^{13}C NMR (126 MHz, DMSO-d_6) δ 15.30 (*C*H_2CH_2), 15.35 (CH_2*C*H_2), 19.33 (*C*$(CH_2)_2$), 35.83 (SCH_2), 36.78 (NHCH*C*H_2), 53.32 (NH*C*HCO), 120.18 (CN), 125.48 (C_{arom}), 126.19 (C_{arom}), 127.54 (C_{arom}), 127.61 (C_{arom}), 128.58 (C_{arom}), 128.71 (C_{arom}), 129.63 (C_{arom}), 132.45 (C_{arom}), 135.76 (C_{arom}), 139.30 (C_{arom}), 167.53 (CONH), 171.49 (CONH). LC-MS (ESI) (60% H_2O to 100% MeOH in 10 min, then 100% MeOH over 10 min, DAD 220-450 nm), 95% purity, *m/z* = 414.0 ($[M+H]^+$), 431.1 ($[M+NH_4]^+$). HRMS (MicroTof QIII), calcd for $C_{21}H_{20}ClN_3O_2S$ 413.10, found 414.1054 (100%), 416.1013 (32%).

Protein Crystallization and Data Collection

Crystals of the SmCB1 complex with **5b** were obtained by vapor diffusion in hanging drop at 5 °C. A drop consisting of 1 µL of the protein solution and 1 µL of the reservoir solution supplemented with 100 µM **5b** was equilibrated over 0.5 mL reservoir solution consisting of 200 mM ammonium acetate, 100 mM sodium citrate, 30% PEG 1500, 2 mM 2-mercaptoethanol, pH 6.2. The obtained needle-shaped crystal was cryoprotected in reservoir solution supplemented with 30% PEG 300, 1.75 mM DTT, 70 µM **5b** before flash-cooling by plunging into liquid nitrogen. Diffraction data were collected at 100 K on MX 14.1 operated by the Joint Berlin MX-Laboratory at the BESSY II electron storage ring in Berlin-Adlershof, Germany.[3] Diffraction data were processed using the XDS suite of programs.[4] Crystal parameters and data collection statistics are given in Table S2.

Structure Determination, Refinement, and Analysis

The SmCB1 complex with **5b** crystallized in the trigonal space group $P3_1$ containing three molecules in the asymmetric unit and solvent content of ~47% (Table S2). The structure of the complex was determined by molecular replacement with the program Molrep[5] from the CCP4 package[6] using the structure of the mature SmCB1 (PDB ID: 4I07)[7] as the search model. Model refinement was performed using the program REFMAC 5.2 from the CCP4 package,[6] interspersed with manual adjustments using Coot.[8] Merohedral twinning was detected and was taken into account during rebuilding and refinement. The structure was refined using data to a resolution of 2.2 Å. The final crystallographic model of SmCB1 contains residues 70–323 (the zymogen numbering). All three molecules in the asymmetric unit are similar: the root-mean-square deviations (RMSDs) for the superposition of the protein backbones ranged from 0.116 to 0.183 Å; these values are within the range observed for different crystal structures of identical proteins.[9] The geometric restraints for **5b** were generated by the program Libcheck[6] using **5b** optimized by the method DFT-D3/B3LYP/DZVP-DFT combined with the COSMO[10] implicit solvent model using the programs Turbomole7.0[11] and Cuby4.[12] The geometric restraints for the covalent link of **5b** were generated by the program Jligand[13,14] using the optimized covalent model (Fig. S7). The molecule of **5b** was modeled with an occupancy of 1 into generally well-defined electron density. The resolution of 2.2 Å does not allow to unambiguously resolve the carbon and boron atom position in the carborane cage. Theoretically, the cage can adopt five possible rotational isomers, differing in the position of the carbon atom (C16) in the upper pentagon of the cage (Fig. S4). The lower value of the electron density of residue Ile145 in the cage proximity indicates some degree of static or dynamic disorder suggesting flexibility of this residue, which may be associated with rotational freedom of the **5b** carborane cage. The final refinement statistics are given in Table S2. The quality of the final model was validated using Molprobity.[15] Atomic coordinates and structure factors were deposited in the Protein Data Bank with accession code 9FZV. Intermolecular interactions were analyzed using the program CONTACT.[6] Molecular graphics in figures were prepared using the program PyMOL 1.4 (Schrödinger).

Enzymatic Assay Materials

Human cathepsin B was purchased from Calbiochem, and human cathepsins K, L, and S from from Enzo. Recombinant cruzain, consisting of the catalytic domain without the C-terminal extension, was expressed and purified as described previously.[16,17] All substrates were purchased from Bachem. Stock solutions of the inhibitors and substrates were prepared in DMSO.

Cathepsin B Inhibition Assay

Kinetic measurements were performed in duplicate in cuvettes (1 mL assay volume) at 37 °C, as described.[18-20] Human cathepsin B was activated by a 1:500 dilution with assay buffer (100 mM sodium phosphate, pH 6.0, 100 mM NaCl, 5 mM EDTA, 0.01% Brij 35) containing 5 mM DTT and then incubated at 37 °C for 30 min. Into a cuvette containing 960 µL assay buffer, 5 µL of the chromogenic substrate Cbz-Arg-Arg-pNA, DMSO and inhibitor solution (15 µL) were pipetted. After addition of cathepsin B (20 µL), substrate hydrolysis was measured spectrophotometrically in a VARIAN/Bio-50/100 photometer at 405 nm for 20 min. The final concentrations of Cbz-Arg-Arg-pNA, cathepsin B and DMSO were 500 µM (= 0.45 × K_m), 19 ng mL^{-1} and 2%, respectively.

Cruzain Inhibition Assay

Kinetic measurements were performed in duplicate with a fluorometric assay (Biotek SynergyTM HT) using Corning 96-well black microplates (200 µL assay volume) at 25 °C. Recombinant cruzain was incubated in 100 mM sodium acetate, pH 5.5, 5 mM DTT, 0.01 % Triton X-100 for 20 min, followed by an additional 2 min incubation with inhibitors before the reaction was started by the addition of the fluorogenic substrate Cbz-Phe-Arg-AMC. Substrate hydrolysis was measured for 5 min fluorometrically at excitation and emission wavelengths of 360 and 460 nm, respectively. Different concentrations of Cbz-Phe-Arg-AMC (Substrate concentrations from 0.2 to 30 µM; Km = 1.4 ± 0.1 µM) were applied, and the final concentration of cruzain and DMSO was 0.15 nM and 5%, respectively.

Cathepsin K Inhibition Assay

Kinetic measurements were performed in duplicate in 96-well microplates (100 µL assay volume) at 25 °C, as described.[18-20] Human recombinant cathepsin K was activated by a 1:100 dilution with assay buffer (100 mM sodium citrate, pH 5.0, 100 mM NaCl, 1 mM EDTA, 0.01% CHAPS) containing 5 mM DTT, and then incubated at 37 °C for 30 min. Into a well containing 194 µL assay buffer, 0.8 µL of the fluorogenic substrate Cbz-Leu-Arg-AMC, DMSO and inhibitor solution (3.2 µL) were pipetted. After addition of cathepsin K (2 µL), substrate hydrolysis was measured for 20 min fluorometrically in a FLUOSTAR Optima plate reader at excitation and emission wavelengths of 360 and 440 nm, respectively. The final concentrations of Cbz-Leu-Arg-AMC, cathepsin K and DMSO were 6 µM (= 3.05 × K_m), 1.73 ng mL^{-1} and 2%, respectively.

Cathepsin L Inhibition Assay

Kinetic measurements were performed in duplicate in cuvettes (1 mL assay volume) at 37 °C, as described.[18-20] Human isolated cathepsin L was activated by a 1:100 dilution with assay buffer (100 mM sodium phosphate buffer, pH 6.0, 100 mM NaCl, 5 mM EDTA, 0.01% Brij 35) containing 5 mM DTT, and then incubated at 37 °C for 30 min. Into a cuvette containing 940 µL assay buffer, 10 µL of the chromogenic substrate Cbz-Phe-Arg-pNA, DMSO and inhibitor solution (10 µL) were pipetted. After the addition of cathepsin L (40 µL), substrate hydrolysis was measured for 20 min spectrophotometrically in a VARIAN/Bio-50/100 photometer at 405 nm. The final concentrations of Cbz-Phe-Arg-pNA, cathepsin L and DMSO were 100 µM (= 5.88 × K_m), 54 ng mL^{-1} and 2%, respectively.

Cathepsin S Inhibition Assay

Kinetic measurements were performed in duplicate in 96-well microplates (100 µL assay volume) at 25 °C, as described.[18-20] Human recombinant cathepsin S was activated by 1:100 dilution with assay buffer (100 mM sodium citrate, pH 6.0 containing 50 mM NaCl, 2 mM EDTA, 0.01% Triton X-100) containing 5 mM DTT, and then incubated at 37 °C for 60 min. Into a well containing 184 µL assay buffer, 0.8 µL of the fluorogenic substrate Cbz-Phe-Arg-AMC, DMSO and inhibitor solution (3.2 µL) were pipetted. After addition of cathepsin S (12 µL), substrate hydrolysis was measured for 20 min fluorometrically on a FLUOSTAR Optima plate reader at excitation and emission wavelengths of 360 and 440 nm, respectively. The final concentrations of Cbz-Phe-Arg-AMC, cathepsin S and DMSO were 40 µM (= 0.74 × K_m), 42 ng mL^{-1} and 2%, respectively.

Supporting Tables

Table S1. Inhibitory affinity of designed compounds and their precursors against selected cathepsin L-type cysteine proteases, including cruzain and human cathepsins K, L, and S.

compound	K_i (µM)[a]			
	cruzain	cat K	cat L	cat S
**5b**	0.12 ± 0.01	0.24 ± 0.03	0.12 ± 0.01	0.59 ± 0.06
**5a**	0.46 ± 0.01	1.41 ± 0.26	0.37 ± 0.02	0.21 ± 0.03
**4**	0.75 ± 0.02	1.85 ± 0.23	0.25 ± 0.02	0.096 ± 0.009

[a] The inhibition constant (K_i) value was measured using a kinetic activity assay with fluorogenic/chromogenic peptide substrates. The standard errors of duplicate measurements refer to the non-linear regression.

Table S2. X-ray data collection and refinement statistics for the SmCB1–**5b** complex

**Data collection statistics**[a]	
Wavelength (Å)	0.918
Temperature (K)	100
Space group	$P3_1$
a, b, c (Å)	81.73, 81.73, 102.09
α, β, γ (°)	90.00, 90.00, 120.00
Resolution (Å)	50.00–2.20 (2.33–2.20)
Number of unique reflections	38651 (6219)
Redundancy	5.9 (5.9)
Completeness (%)	99.8 (99.6)
R_{merge}[b] (%)	28.0 (187.4)
Average I/σ (I)	5.21 (0.76)
$CC_{1/2}$ (%)	98.5 (35.2)
Wilson B (Å^2)	38.5
**Refinement statistics**	
Resolution range (Å)	41.40–2.20 (2.26–2.20)
Number of reflections in working set	36677 (2728)
Number of reflections in test set	1972 (120)
R value[c] (%)	20.2 (25.4)
R_{free} value[d] (%)	25.0 (31.7)
Number of molecules in AU[e]	3
Number of atoms in AU[e] protein/inhibitor/solvent	5999/102/117
Average ADP[f] for protein/inhibitor/solvent (Å^2)	38.9/30.5/33.9
RMSD bond length (Å)	0.013
RMSD bond angle (°)	1.44
Ramachandran plot statistics[g]	
Favored regions (%)	95.1
Allowed regions (%)	4.9
PDB code	9FZV

[a] Numbers in parentheses refer to the highest-resolution shell.

[b] $R_{merge}=100\sum_{hkl}\sum_i|I_i(hkl) - \langle I(hkl)\rangle|/\sum_{hkl}\sum_i I_i(hkl)$, where $I_i(hkl)$ is an individual intensity of the ith observation of the reflection *hkl* and $\langle I(hkl)\rangle$ is the average intensity of the reflection *hkl* with summation over all data.

[c] R value = $||F_o| - |F_c||/|F_o|$, where F_o and F_c are the observed and calculated structure factors, respectively.

[d] R_{free} is equivalent to the R value but is calculated for up to 5% of the reflections chosen at random and omitted from the refinement process.[21]

[e] AU, asymmetric unit.

[f] ADP, atomic displacement parameter, formally B-factor.

[g] As determined by Molprobity.[15]

Table S3. QM/MM relative 'free' energy of the studied complexes, with and without C-H···O-C H-bond between the inhibitor and the Glu142 side chain in kcal mol^{-1}, see also Fig. S6

Complex	Relative 'free' energy	
	Complex with the H-bond	Complex without the H-bond
SmCB1–**5b**	0.0	0.7
SmCB1–**5a**	3.4	0.0

Table S4. The QM/MM-based protein–ligand score computed as a sum of following term: 'free' energy difference between covalent and noncovalent complexes ($\Delta G'_{cov}$), interaction energy (ΔE), interaction solvation free energy ($\Delta\Delta G_{solv}$), and change of conformational 'free' energy ($\Delta G'_{conf}$). All in kcal mol^{-1}

Complex	$\Delta G'_{cov}$	ΔE	$\Delta\Delta G_{solv}$	$\Delta G'_{conf}$.	Total
SmCB1–**5b**	-25.5	-128.6	62.7	17.8	-73.7
SmCB1–**5a**	-21.0	-107.9	46.9	11.3	-70.8

Table S5. The results of interaction energy decomposition into electrostatic (E_{elec}), induction (E_{ind}), dispersion (E_{disp}) and exchange (E_{exch}) contributions by using the SAPT0/jun-cc-pVDZ methodology. All energies are in kcal mol^{-1}. The relative values in parentheses show the contribution to the sum of all the attractive energy terms

	Total	E_{elec}	E_{exc}	E_{ind}	E_{disp}
**5b**:Gly138-Leu139	-3.50	-0.76 (10.1%)	4.03	-0.80 (10.6%)	-7.53 (79.3%)
**5b**:Glu142	-22.29	-18.71 (51.0%)	14.39	-9.57 (26.1%)	-8.40 (22.9%)
**5b**:Gly142-Gly144-Ile145	-7.18	-3.10 (23.4%)	6.08	-1.05 (7.9%)	-9.11 (68.7%)
sum	-32.97	-22.58	24.50	-11.42	-23.48
**5a**:Gly138-Leu139	-3.78	-2.25 (24.8%)	5.28	-0.79 (8.8%)	-6.01 (66.4%)
**5a**:Glu142	-0.65	0.42	1.44	-0.70 (28.0%)	-1.81 (72.0%)
**5a**:Gly143-Gly144-Ile145	-6.24	-3.36 (28.5%)	5.55	-0.90 (7.6%)	-7.53 (63.9%)
sum	-10.67	-5.19	12.26	-2.40	-23.36

Table S6. Decomposition of interaction energies using the SAPT0/jun-cc-pVDZ methodology for the carborane cage linked via CH functionalization, see Fig. S8. All energies are in kcal mol^{-1}

Interaction with	Total	E_{elec}	E_{exc}	E_{ind}	E_{disp}
Gly138-Leu139	-2.62	-0.59 (9.2%)	3.86	-0.64 (9.9%)	-5.24 (81.0%)
Glu142	-3.74	1.61	7.57	-5.64 (43.7%)	-7.27 (56.3%)
Gly142-Gly144-Ile145	-7.60	-3.05 (24.7%)	4.78	-0.97 (7.8%)	-8.35 (67.5%)
sum	-13.96	-2.04	16.20	-7.25	-31.76

Supporting Figures

A

Compound	Phenotype descriptors[a]						Severity score[b]					
	1 µM			10 µM			1 µM			10 µM		
	Day 1	Day 2	Day 3	Day 1	Day 2	Day 3	Day 1	Day 2	Day 3	Day 1	Day 2	Day 3
**1**	N	N	N	N	N	N	0	0	0	0	0	0
**2**	N	N	N	N	N	N	0	0	0	0	0	0
**3**	N	N	N	N	N	N	0	0	0	0	0	0
**4**	N	N	N	N	N	N	0	0	0	0	0	0
**5a**	N	N	N	N	N	N	0	0	0	0	0	0
**5b**	N	Dark	Dark, R	N	Dark, R	Deg	0	1	2	0	2	4

[a]Phenotypes were reported using the following descriptors: N, normal; R, rounded; S, slow; unc, uncoordinated; Dark, dark (color altered from normal); Deg, degenerated. [b]Each descriptor is assigned a value of 1, except for Deg, which is given the maximum value of 4. Values are then added to yield a severity score ranging from 0 (no effect) to 4 (the most severe), as described previously.[22-25] Compounds were tested in duplicate in two independent assays (representative data are shown).

B

Compound	Cell line viability after 72 h (%)							
	CCRF-CEM		HL-60		HeLa		HepG2	
	1 µM	10 µM	1 µM	10 µM	1 µM	10 µM	1 µM	10 µM
**1**	99	98	100	98	96	98	98	103
**2**	91	89	107	95	95	95	95	97
**3**	92	84	102	103	93	90	93	103
**4**	99	96	94	97	87	94	100	99
**5a**	93	102	96	97	112	102	100	99
**5b**	99	93	101	103	94	90	92	96

Fig. S1. Anti-schistosomal activity and cytotoxicity of compounds. **A**. Phenotypic changes in newly transformed schistosomula (NTS) of *S. mansoni* induced by 1 and 10 µM compounds were recorded daily for three days. **B**. Cytotoxicity of the compounds towards six human cell lines expressed as % viability *vs*. untreated cells. Cells were treated with the indicated concentration of the compounds for 72 h and viability measured by means of the XTT assay, as described previously.[25,26] All measurements were performed in triplicate.

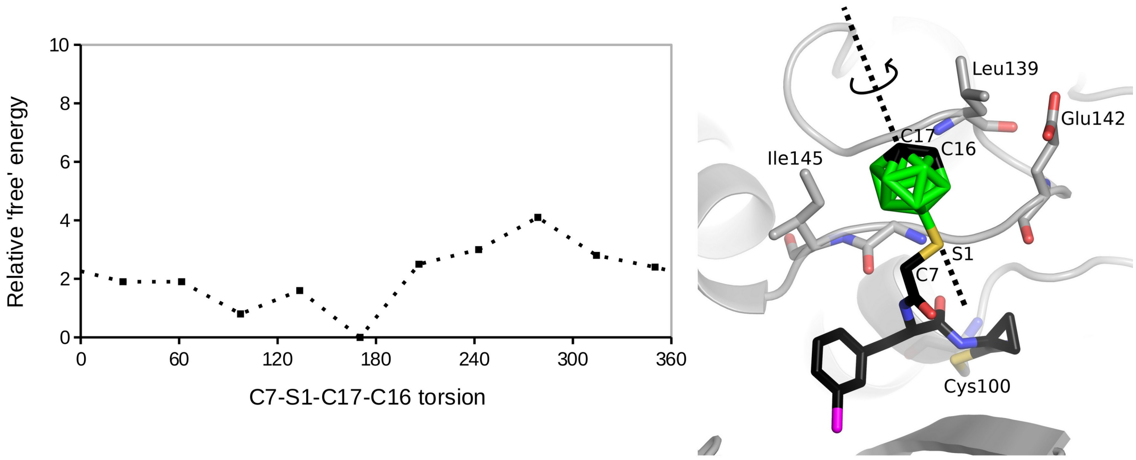

Fig. S2. Relative ‘free’ energy plotted against the C7-S1-C17-C16 torsion. Energy is in kcal mol^{-1} and torsion in degrees. The C16 is oriented towards Glu142, optimizing the orientation of the dipole moment of the carborane cage towards the charge group. The rotational profile of the carborane moiety in the SmCB1–**5b** complex highlights the fact that the rotamers are distinguished only by the positions of the BH and CH groups. Distinguishing the carbon and boron vertices is an established problem in structural studies of carborane because the atomic scattering factors for X-rays of carbon and boron are similar due to their adjacency in the periodic table.[27]

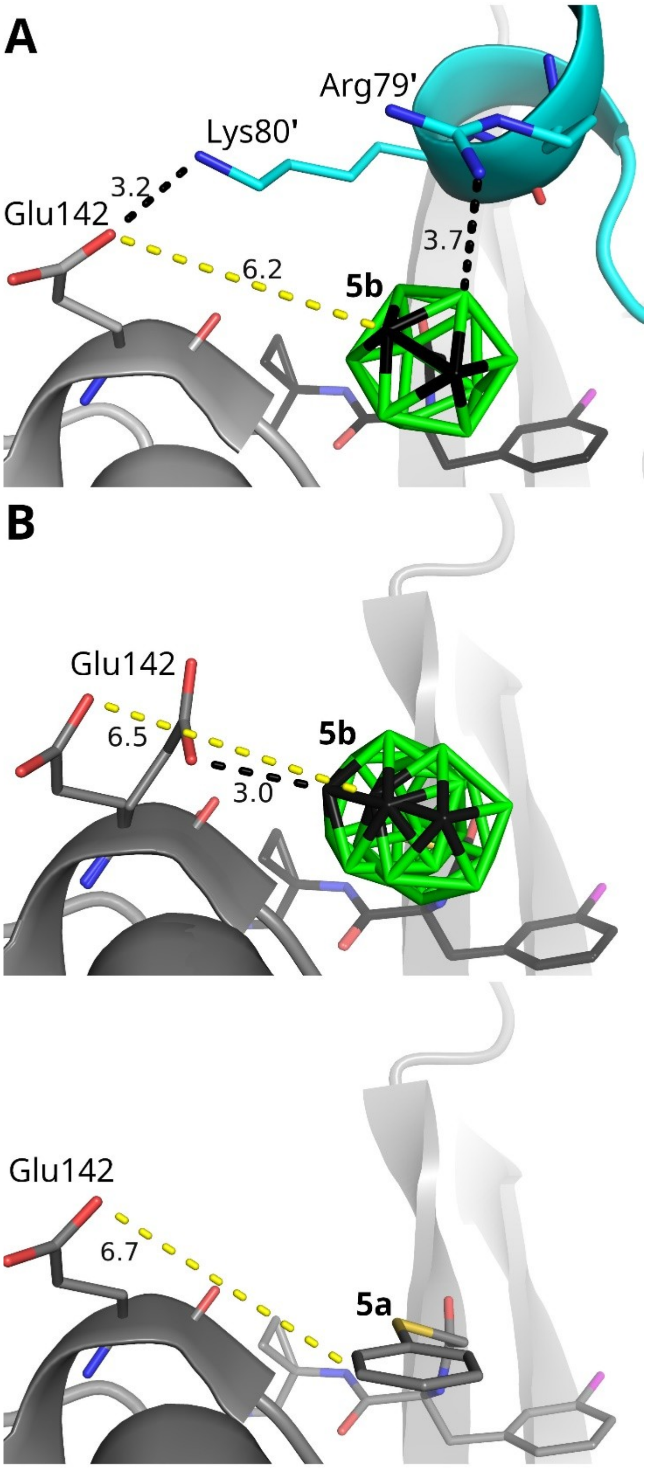

Fig. S3. Glu142 residue in the active site of the SmCB1–**5b** crystal structure and optimization of its side-chain position in the SmCB1–**5b** and the SmCB1–**5a** QM/MM models. **A**. Crystal structure of the SmCB1–**5b** complex (9FZV). The S3 subsite of SmCB1 (shown as gray cartoon), which accommodates the carborane cage of **5b** (stick representation; C atoms in black), is partially solvent-exposed and may be influenced by the symmetry-related molecule (in cyan, residues labeled with an apostrophe). H-bonds stabilizing the SmCB1–**5b** complex in the crystal are depicted as black dashed lines. Namely, the B1 atom of the carborane cage forms a diH-bond with Arg79' and the Glu142 residue forms a H-bond with Lys80', both from the symmetry-related molecule. For illustration, distances measured without H-bonds are depicted as yellow dashed lines. Distance values are in Å. Heteroatoms have a standard color coding (B – green; O – red; N – blue; Cl – magenta; S – yellow).

B. The QM/MM model of the SmCB1–**5b** (upper panel) and the SmCB1–**5a** (lower panel) complexes (carbon atoms are in gray) constructed from the SmCB1–**5b** crystal structure (panel A) using QM/MM computations that exclude the influence of symmetry-related molecules. Upon unrestrained QM/MM optimization, the C/**5b**···O/Glu142 distance increased from 6.2 to 6.5 Å However, the relaxed scan along the CH/**5b**···OE1/Glu142 H-bond pathway yielded a more favorable model in which the H-bond is formed between the inhibitor and the Glu142 side chain (see Table S3). Hydrogen atoms are omitted for clarity. This H-bond was energetically favorable in the SmCB1–**5b** model but not in the SmCB1–**5a** model (not shown; see Table S3).

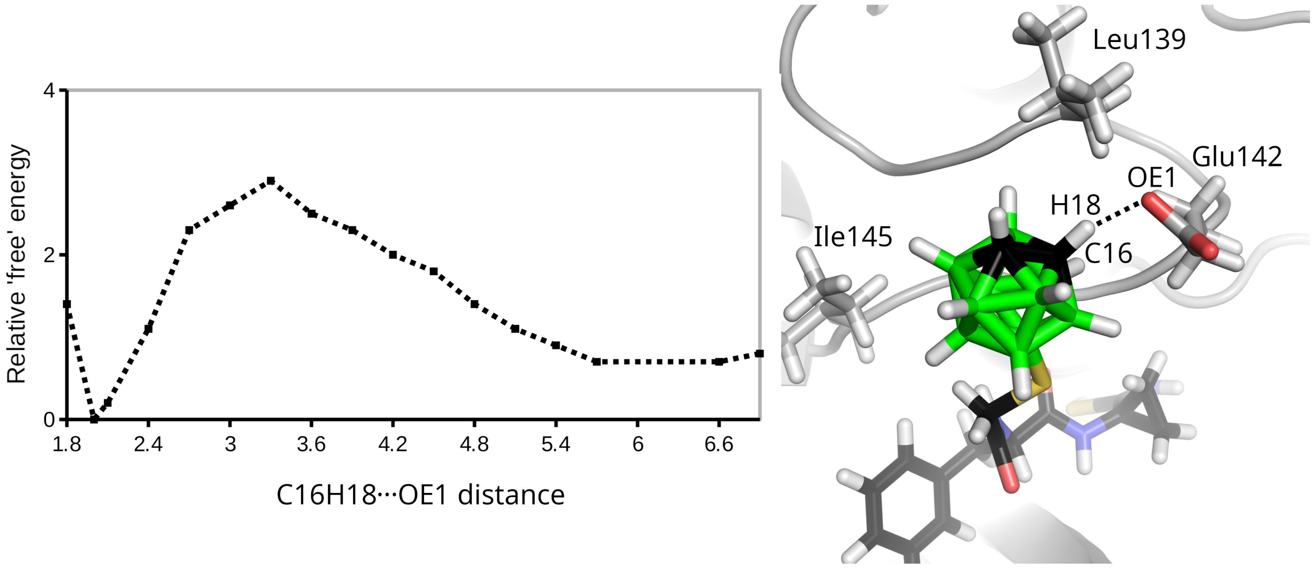

Fig. S4. Results of the computed relaxed scan along the CH/**5b**···OE1/Glu142 H-bond. The relative 'free' energy is plotted against the C16H18/**5b**····OE1/Glu142 distance. Energy is in kcal mol^{-1} and distance in Å.

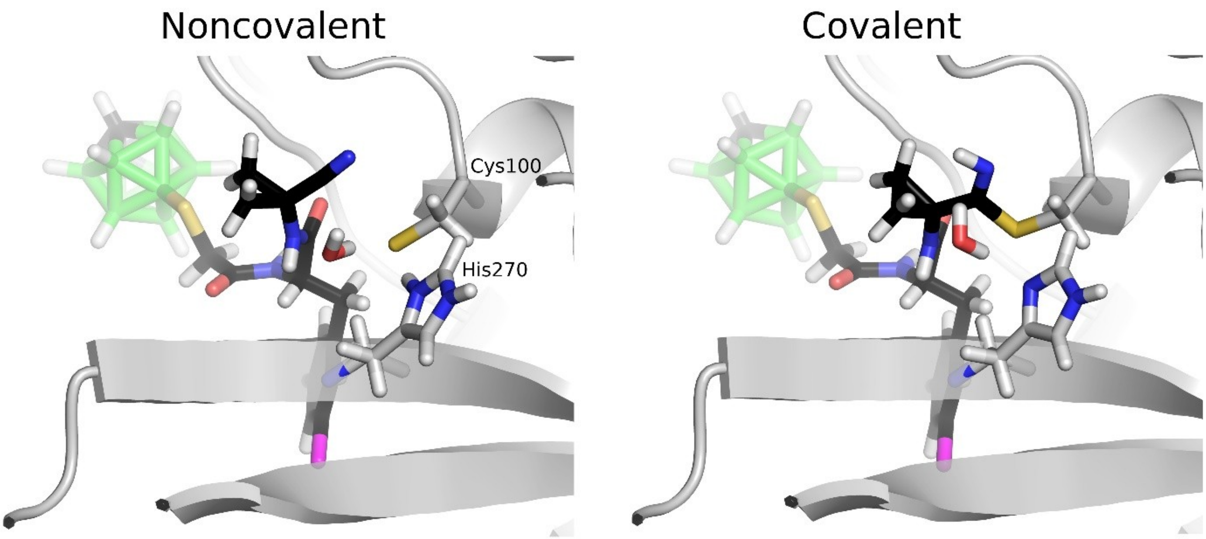

Fig. S5. Structural details of the modeled noncovalent SmCB1–**5b** complex (left) and the covalent SmCB1–**5b** complex (right).

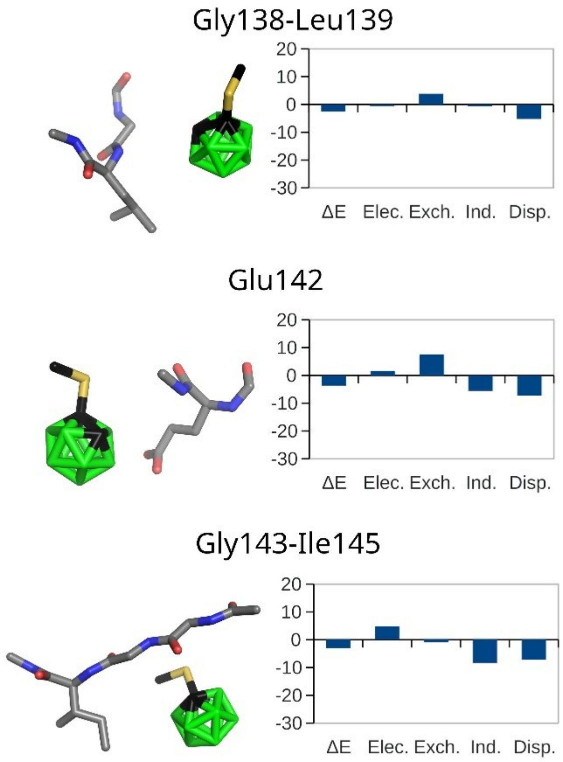

Fig. S6. Decomposition of ΔE between the C(1)-linked carborane cage and the surroundings amino acids of the SmCB1 S3 subsite into electrostatic (Elec.), induction (Ind.), dispersion (Disp.) and exchange (Exch.) contributions. Energies are in kcal mol^{-1}.

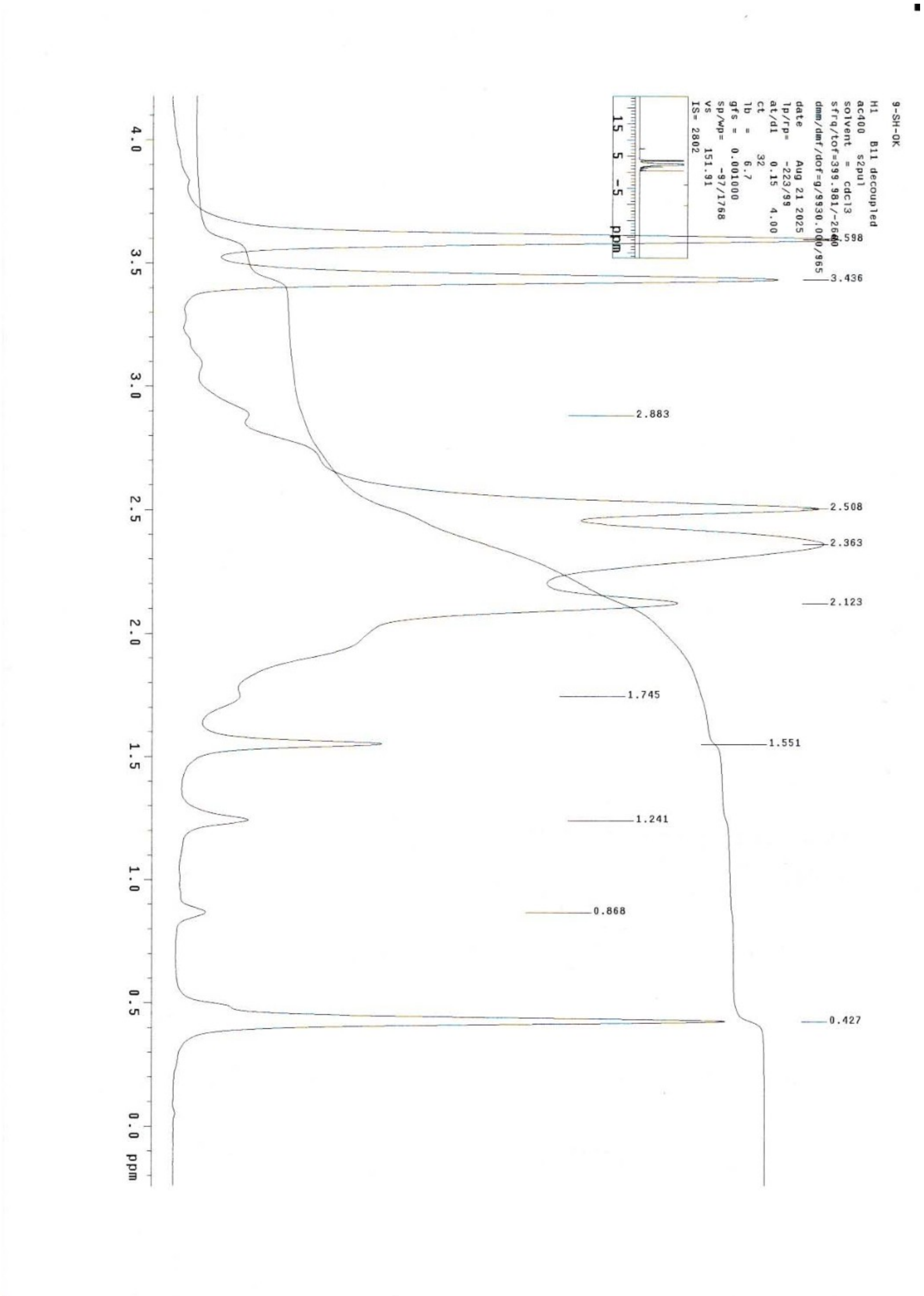

Fig. S7. ^{1}H NMR spectrum (400 MHz, $CDCl_3$) of precursor **2**: **δ 0.42 – 2.88 (m, 10H,** B-H**,** SH**), 3.43 (s, 1H,** C2-H**), 3.59 (s, 1H,** C1-H**),** for details see Syntheses and Characterizations.

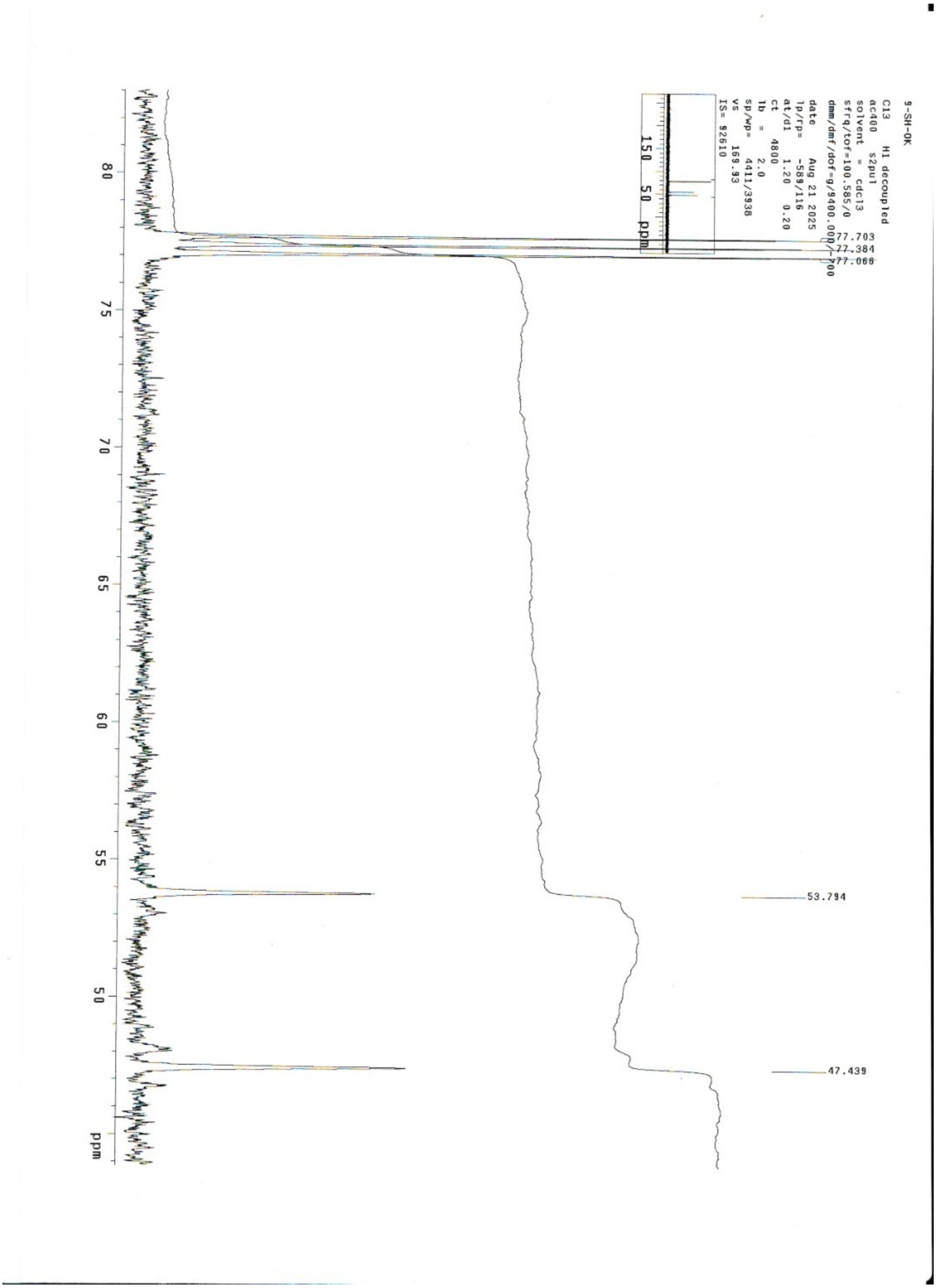

Fig. S8. ^{13}C NMR spectrum (400 MHz, $CDCl_3$) of precursor **2**: δ 47.43 (**C1**), 53.79 (**C2**), for details see Syntheses and Characterizations.

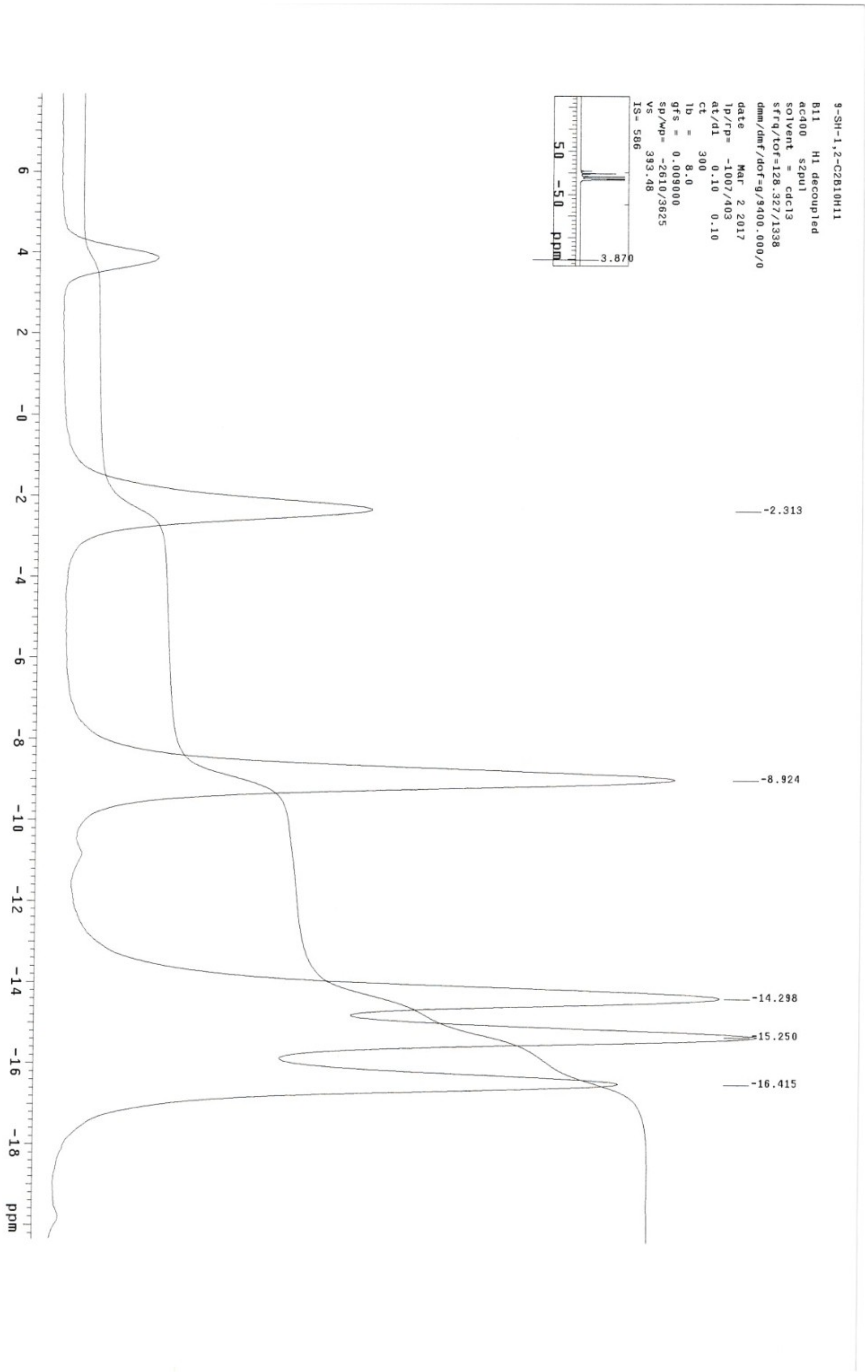

Fig. S9. ^{11}B NMR spectrum (400 MHz, $CDCl_3$) of precursor **2:** δ -16.4 (2d, **B3,6**), -15.3 (2d, **B7,11**), -14.3 (2d, **B4,5**), -8.9 (2d, **B8,10**), -2.3 (1d, **B12**), 3.9 (1s, **B9**), for details see Syntheses and Characterizations, systematic carborane numbering is provided in Fig. S26.

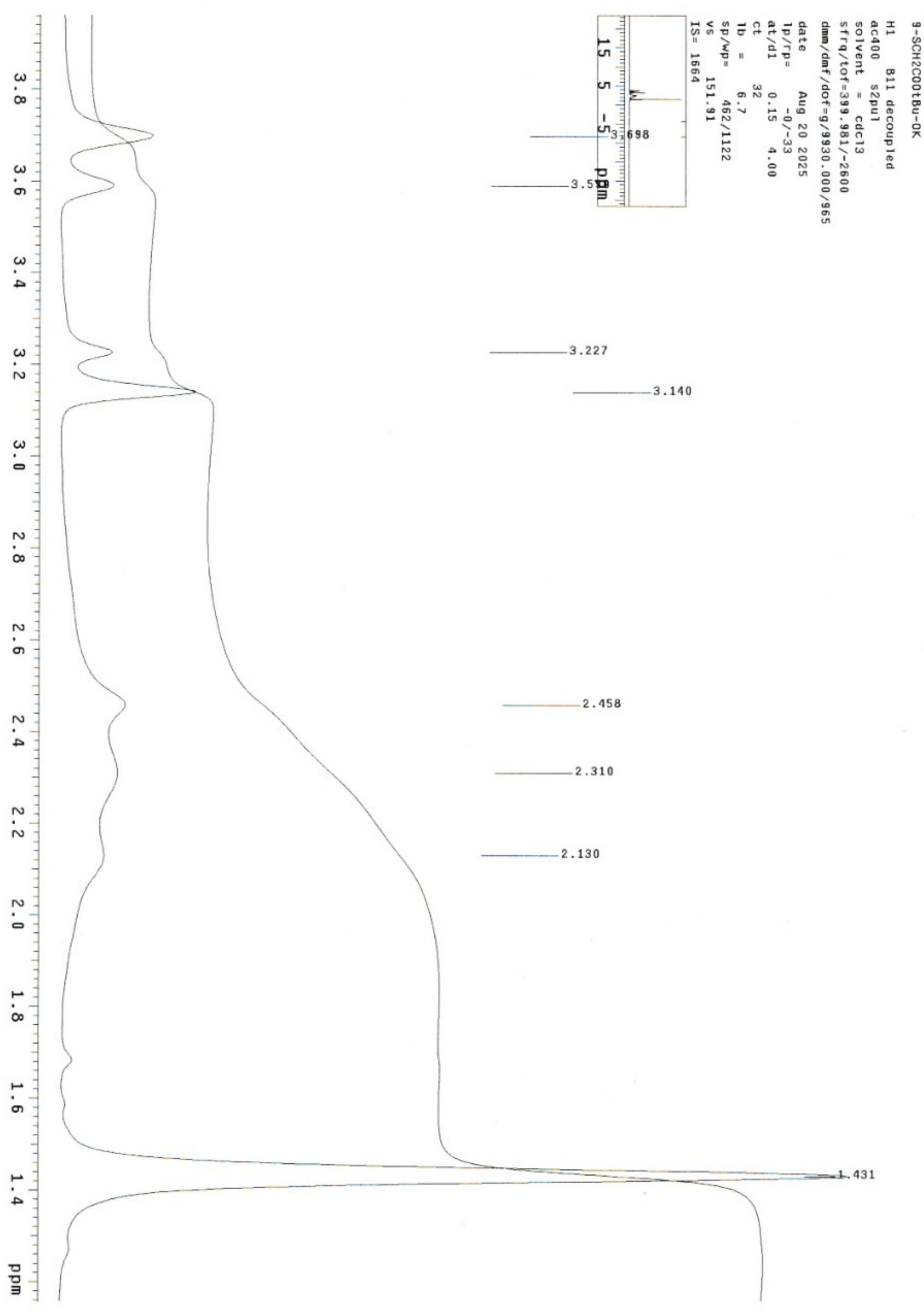

Fig. S10. ^{1}H NMR spectrum (400 MHz, $CDCl_3$) of precursor **3:** δ 1.43 (s, 9H, **$CO(CH_3)_3$**), 2.13 – 2.45 (m, 9H, **B_9H_9**), 3.14 – 3.22 (m, 2H, **CH_2**), 3.56 – 3.69 (m, 2H, **C2-H**, **C1-H**), for details see Syntheses and Characterizations.

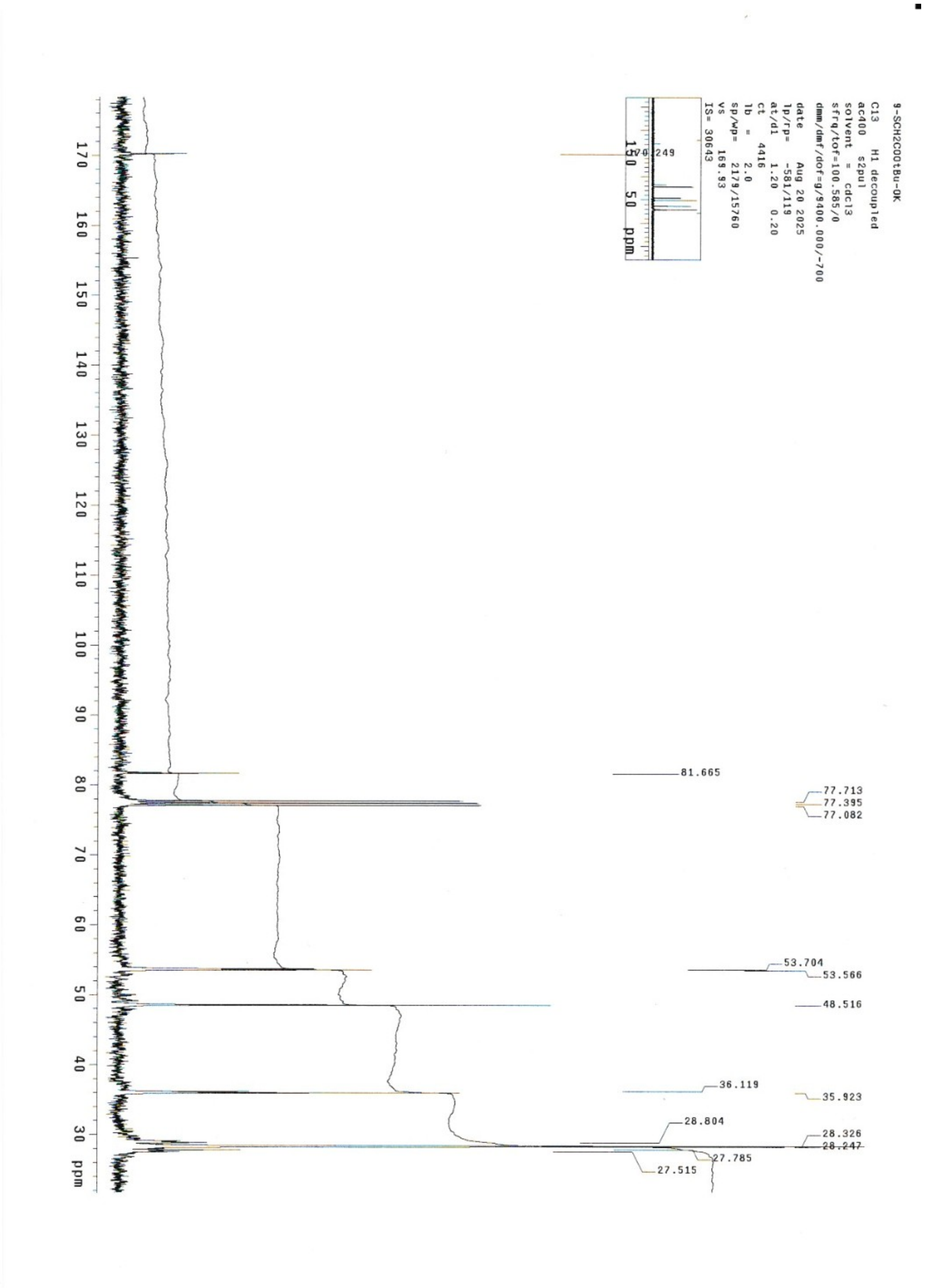

Fig. S11. ^{13}C NMR spectrum (400 MHz, $CDCl_3$) of precursor **3**: δ 28.33 (($CO_2C($***CH_3***$)_3$), 35.92 (**CH_2**), 48.52 (**C1**), 53.70 (**C2**) 81.67 (CO_2*C*$(CH_3)_3$), 170.25 (***CO***$_2C(CH_3)_3$), for details see Syntheses and Characterizations.

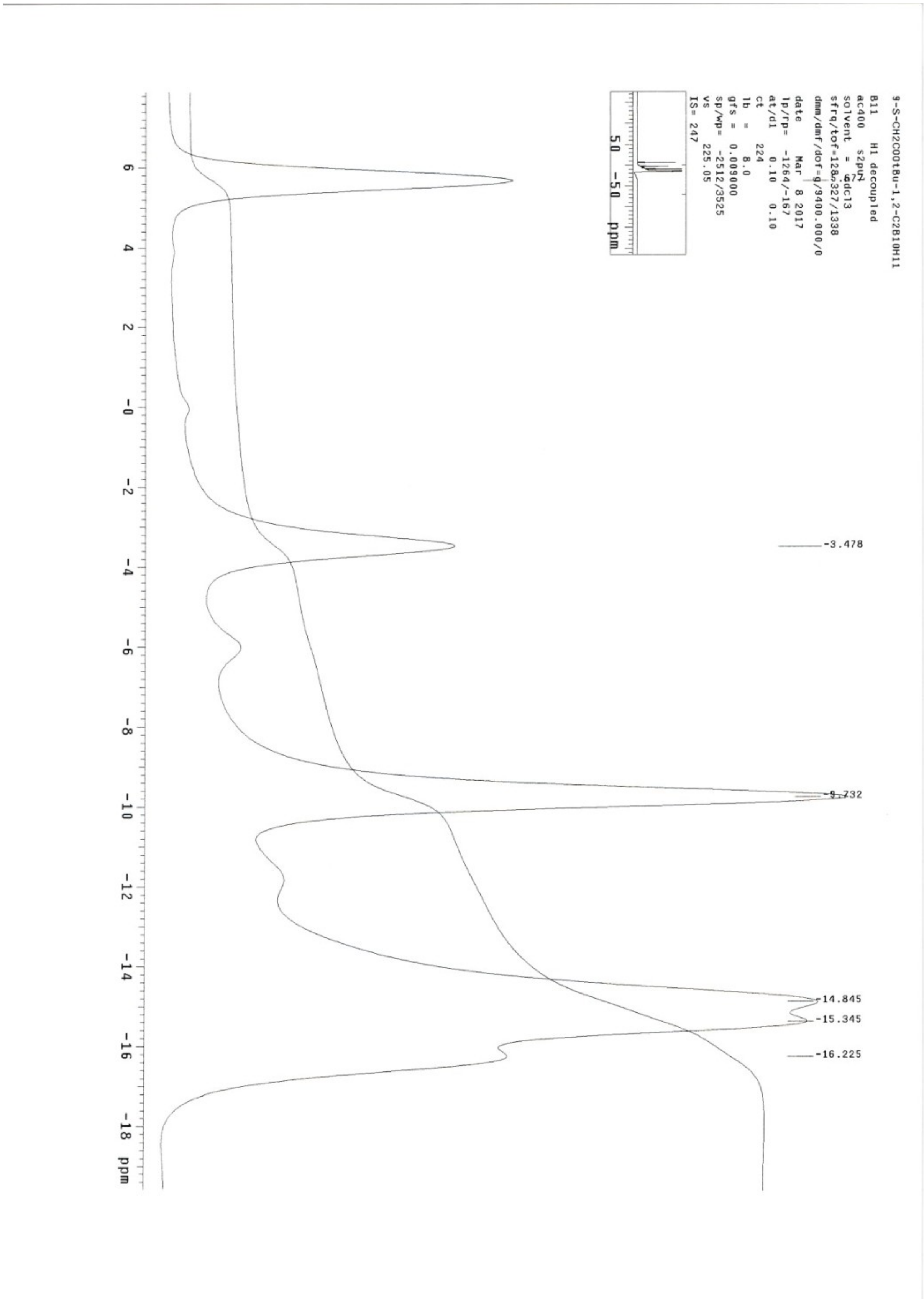

Fig. S12. ^{11}B NMR spectrum (400 MHz, $CDCl_3$) of precursor **3:** δ -16.2 (2d, **B3,6**), -15.3 (2d, **B7,11**), -14.8 (2d,**B4,5**), -9.7 (2d, **B8,10**), -3.5 (1d, **B12**, 5.7 (1s, **B9**), for details see Syntheses and Characterizations, systematic carborane numbering is provided in Fig. S26.

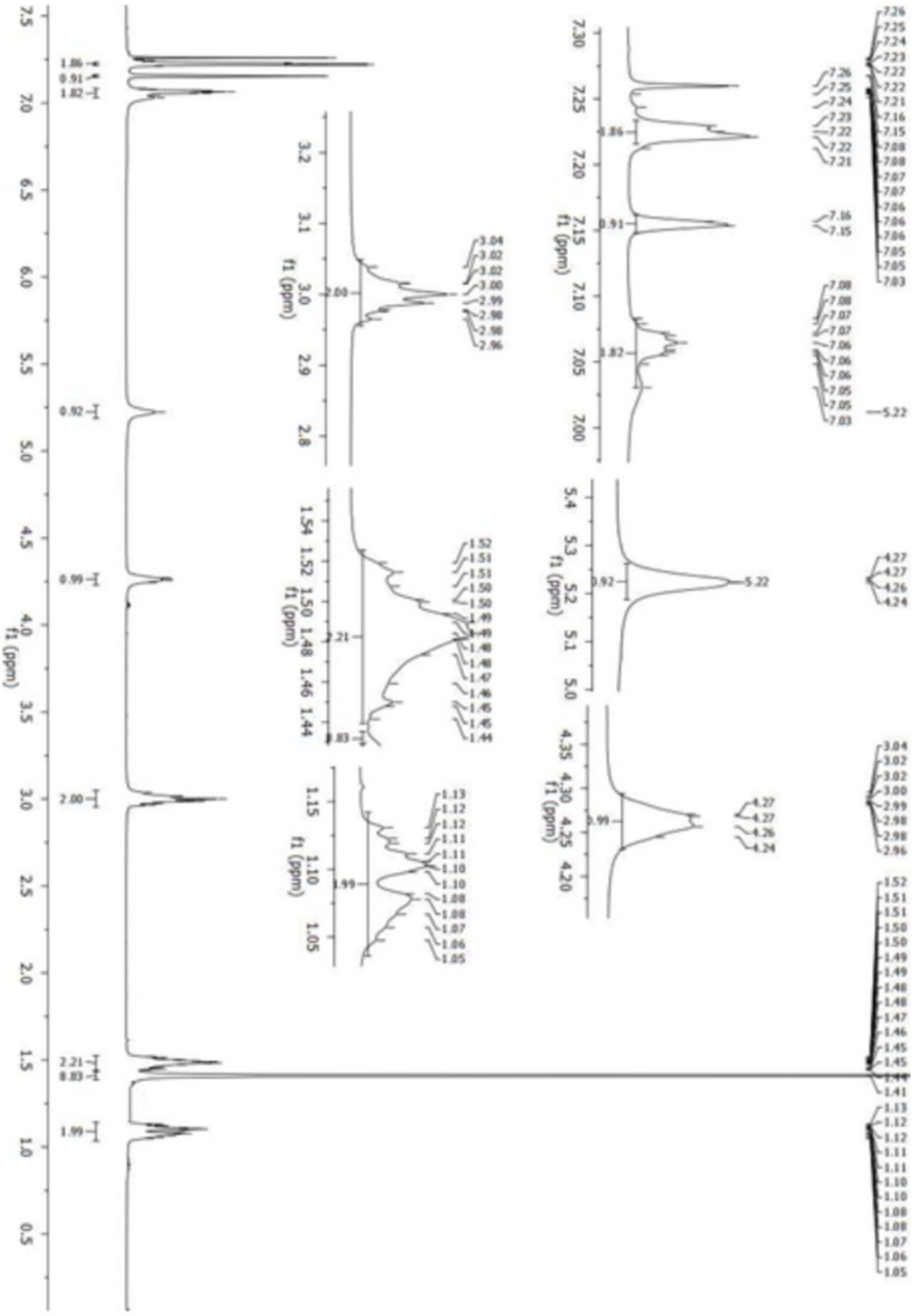

Fig. S13. ^{1}H NMR spectrum (500 MHz, $CDCl_3$) of compound **4**: δ 1.05 – 1.13 (m, 2H, **CH_2CH_2**), 1.41 (s, 9H, **C(C*H*$_3$)$_3$**), 1.44 – 1.52 (m, 2H, **CH_2CH_2**), 2.96 – 3.04 (m, 2H, **NHCHC*H*$_2$**), 4.25 (br s, 1H, **NHC*H*CH_2**), 5.22 (br s, 1H, **CON*H*CH**), 7.03 – 7.08 (m, 2H, **H_{arom}**), 7.16 (br s, 1H, **H_{arom}**), 7.22 (br s, 1H, **H_{arom}**), 7.23 (br s, 1H, **CON*H*C(CH_2)$_2$**), for details see Syntheses and Characterizations

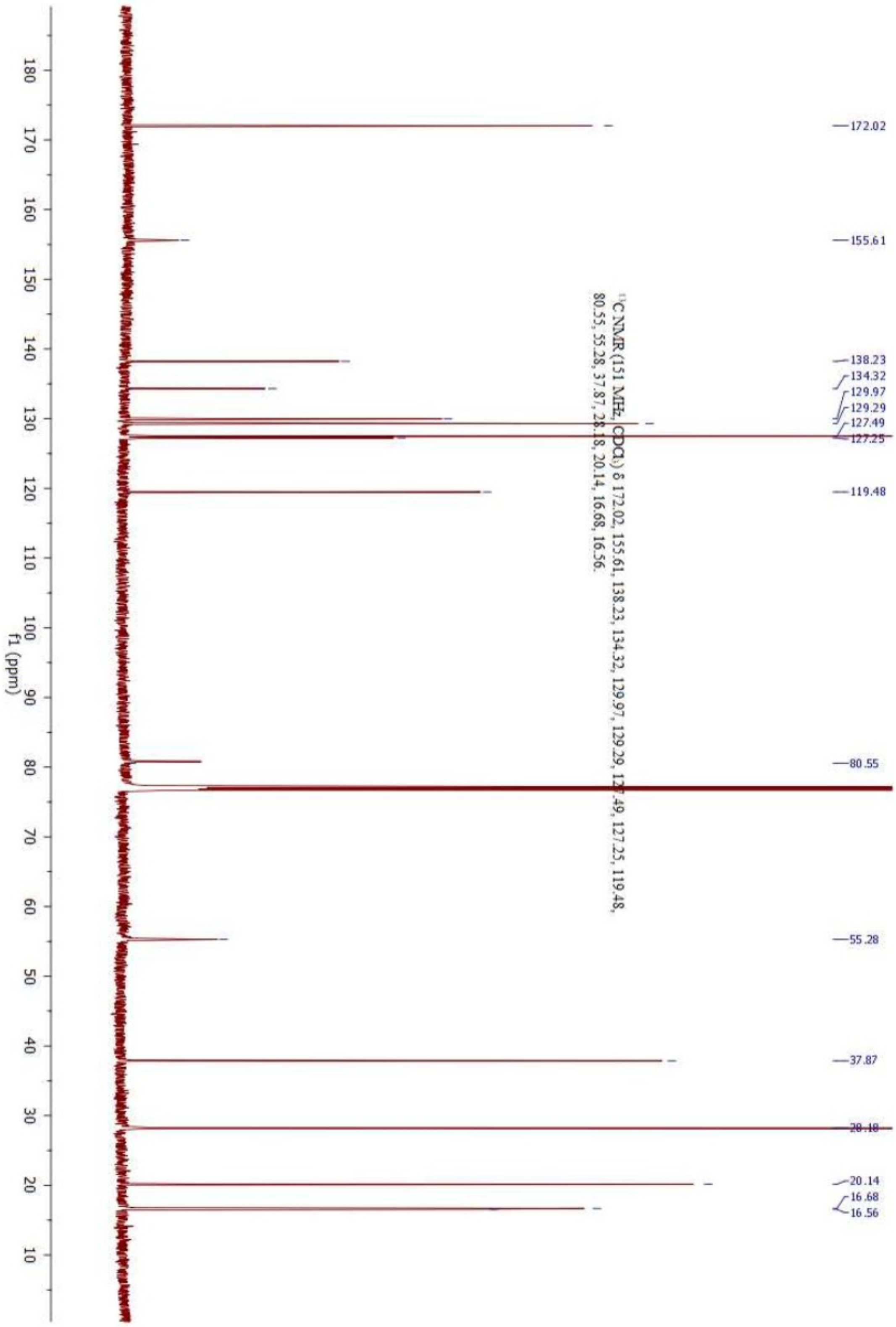

Fig. S14. ^{13}C NMR spectrum (151 MHz, $CDCl_3$) of compound **4**: δ 16.56 (**CH_2CH_2**), 16.68 (**CH_2CH_2**), 20.14 (**$C(CH_2)_2$**), 28.18 (**$CO_2C(CH_3)_3$**), 37.87 (**$NHCHCH_2$**), 55.28 (1C, **NHCHCO**), 80.55 (**$CO_2C(CH_3)_3$**), 119.48 (**CN**), 127.25 (**C_{arom}**), 127.49 (**C_{arom}**), 129.29 (**C_{arom}**), 134.32 (**C_{arom}**), 138.23 (**C_{arom}**), 155.61 (**$NHCO_2C(CH_3)_3$**, 172.02 (**CHCONH**), for details see Syntheses and Characterizations

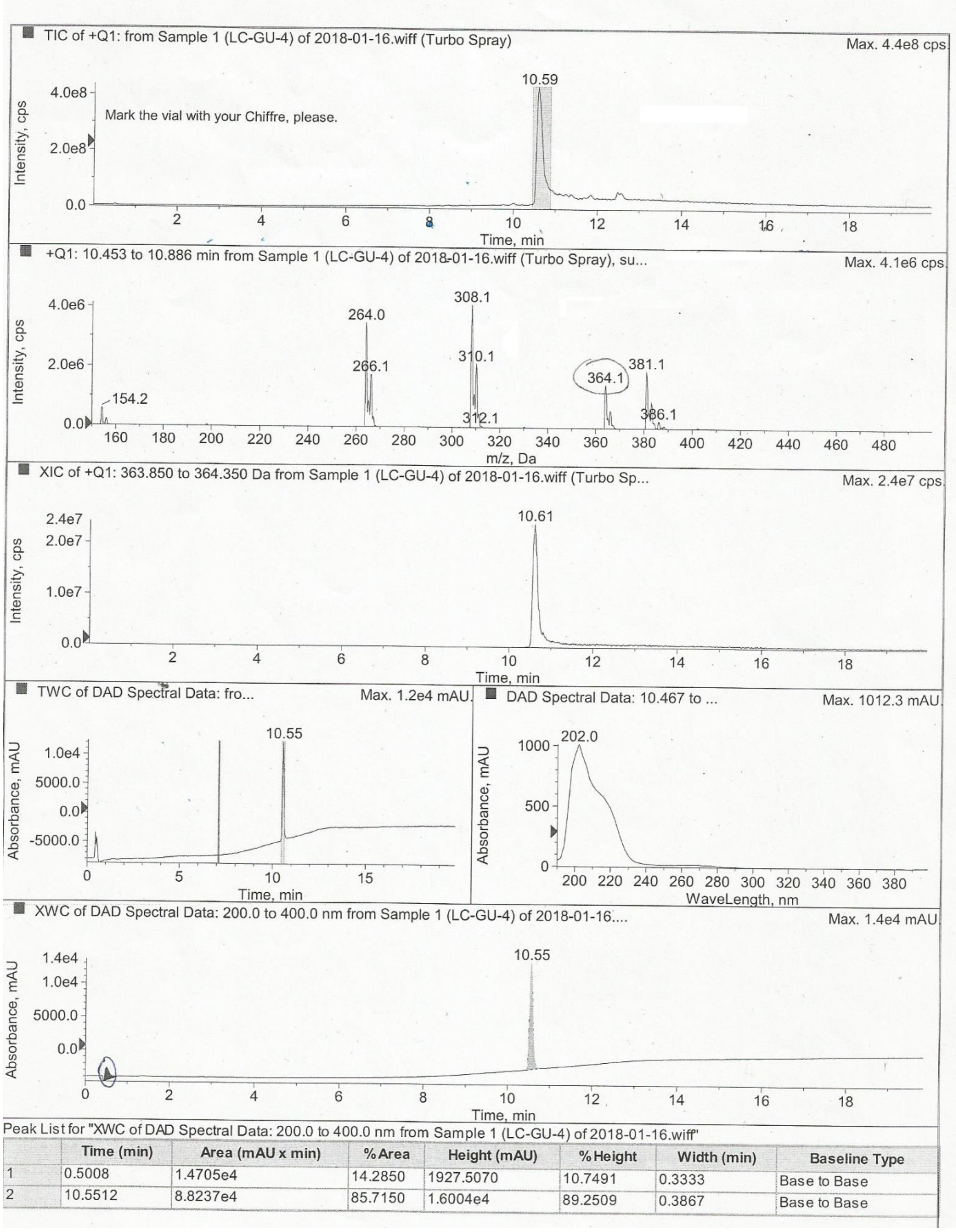

Peak List for "XWC of DAD Spectral Data: 200.0 to 400.0 nm from Sample 1 (LC-GU-4) of 2018-01-16.wiff"

	Time (min)	Area (mAU x min)	%Area	Height (mAU)	%Height	Width (min)	Baseline Type
1	0.5008	1.4705e4	14.2850	1927.5070	10.7491	0.3333	Base to Base
2	10.5512	8.8237e4	85.7150	1.6004e4	89.2509	0.3867	Base to Base

Fig. S15. HPLC–MS chromatogram of compound **4**. Peak at R_t 0.50 min: solvent peak; peak at R_t 10.55 min: compound **4** with detected mass of 364.1 $[M+H]^+$.

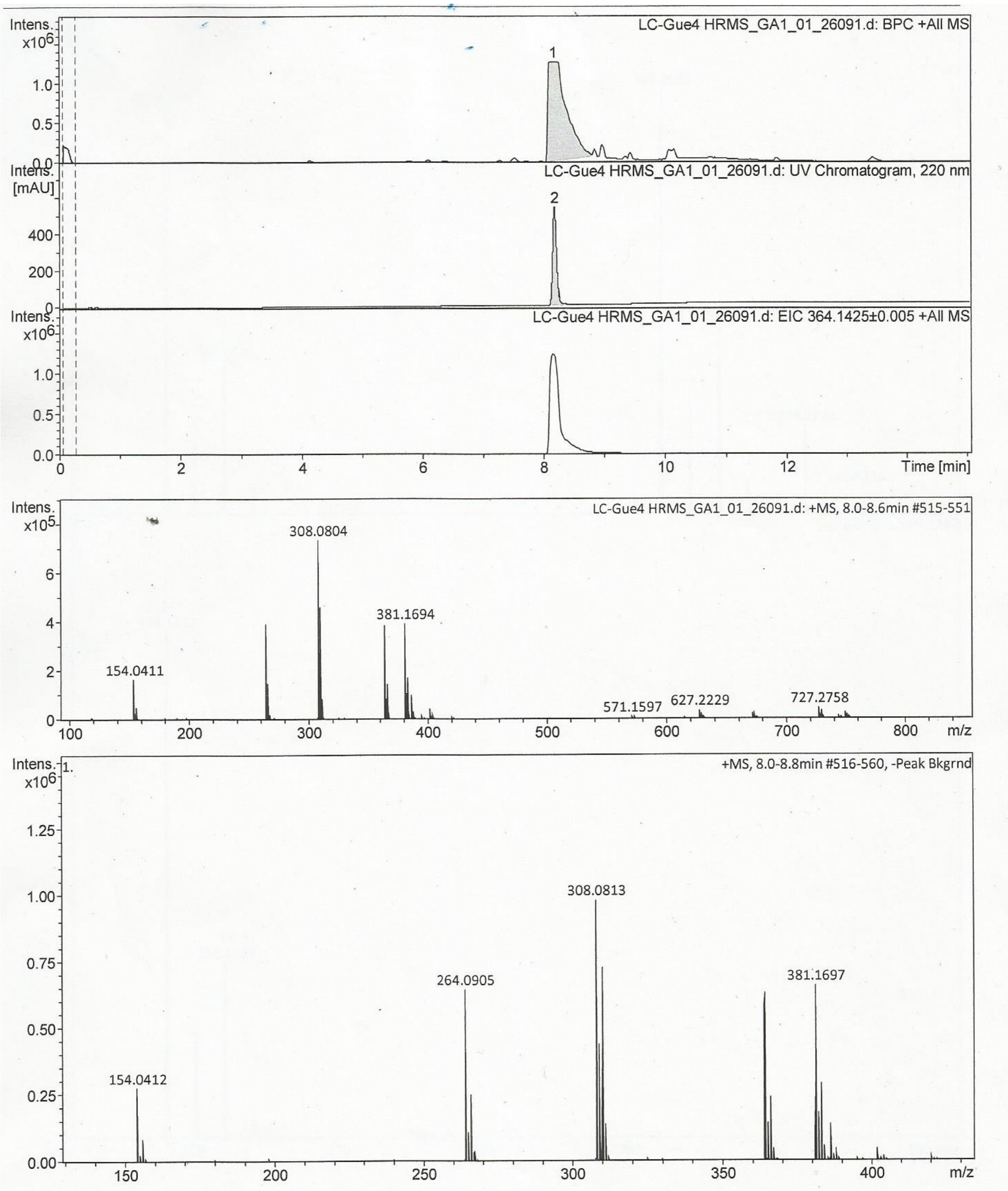

Fig. S16. HPLC-HRMS (micrOTOF-QIII) spectrum of compound **4**; observed *m/z* corresponds to the expected molecular ions: $[M+H]^+$ = 364.1425 (100%), 366.1399 (32%); $[M+NH_4]^+$ = 381.1697; $[M\text{-}Boc]^+$ = 264.0905.

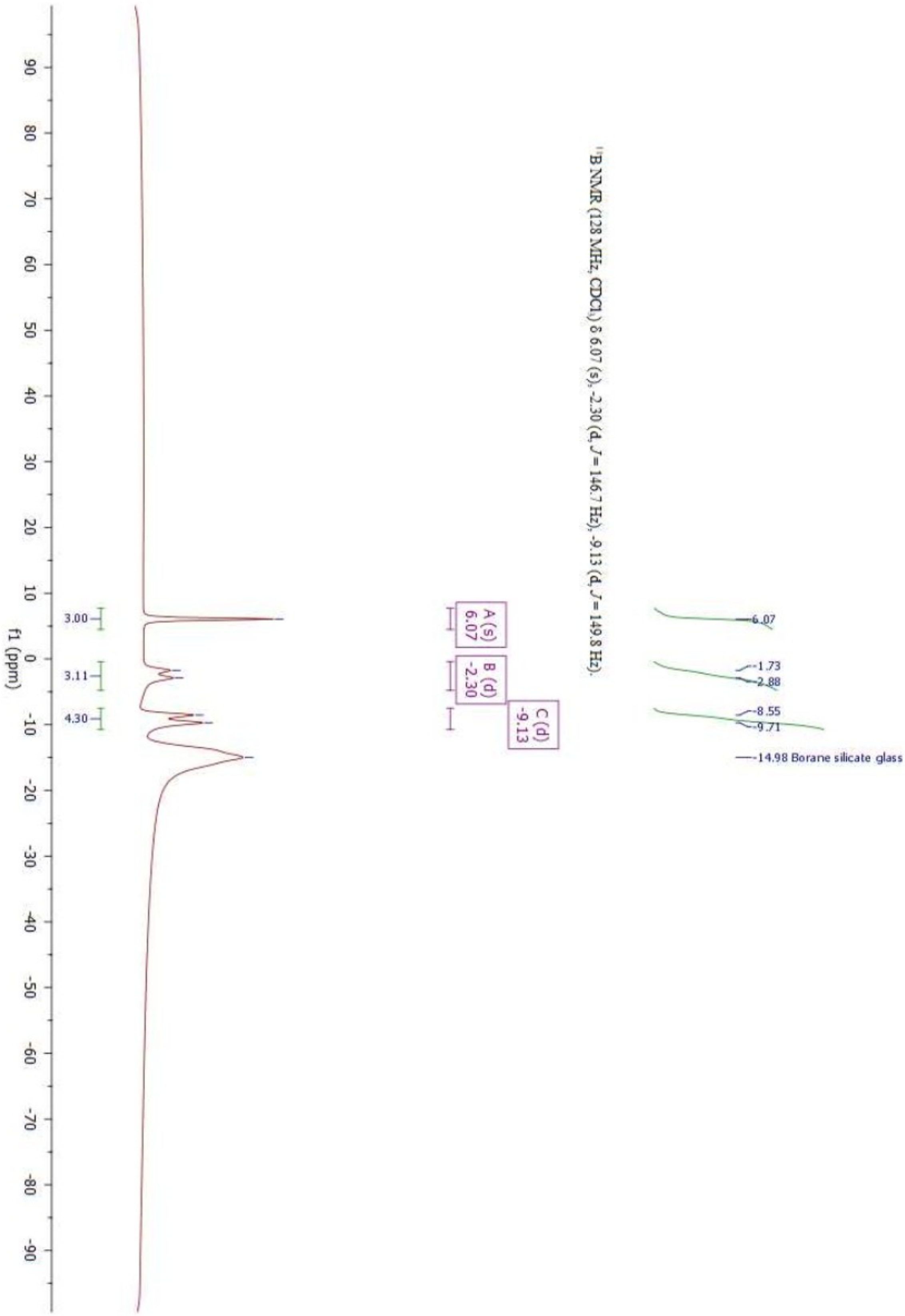

Fig. S17. ^{11}B NMR spectrum (128 MHz, $CDCl_3$) of compound **5b**: ^{11}B NMR (128 MHz, $CDCl_3$), ^{1}H-decoupled) δ -15.0 (m, **B3,6,4,5,7,11**), -2.9 (2d, **B8,10**), -1.7 (1d, **B12)**, 6.1 (1s, **B9**)., for details see Syntheses and Characterizations, systematic carborane numbering is provided in Fig. S26.

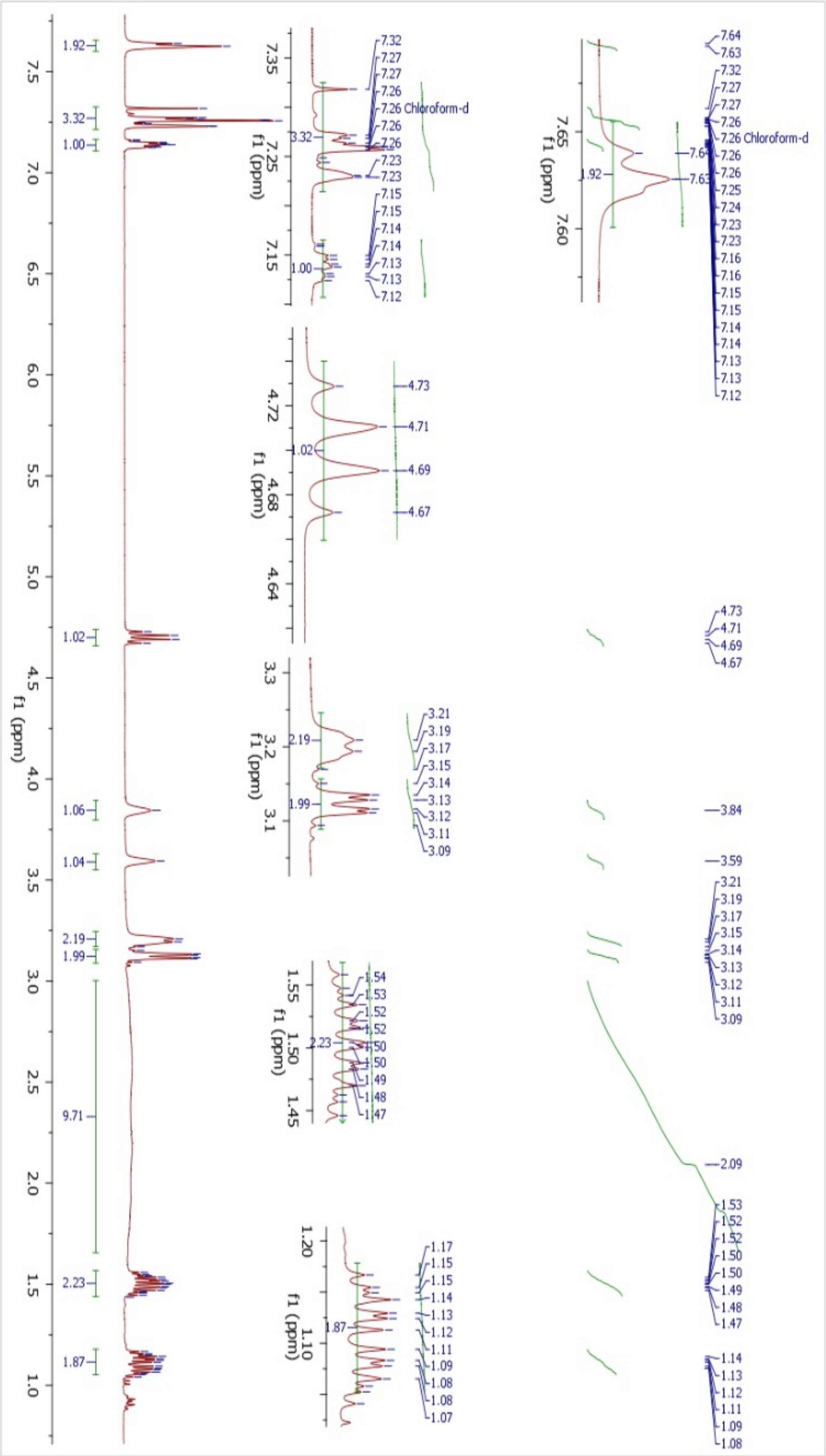

Fig. S18. ^{1}H NMR spectrum (500 MHz, $CDCl_3$) of compound **5b**: δ 1.05 – 1.18 (m, 2H, **CH_2CH_2**), 1.44 – 1.57 (m, 2H, **CH_2CH_2**), 1.52 – 3.09 (m, br, 9H, **B_9*H_9***), 3.09 – 3.14 (m, 2H, **SCH_2**), 3.17 – 3.21 (m, 2H, **NHCHC*H_2***), 3.59, 3.84 (each s, each 1H, **C2-*H***, **C1-*H***), 4.67 – 4.73 (m, 1H, **NHC*H*CH_2**), 7.11 – 7.17 (m, 1H, **H_{arom}**), 7.21 – 7.33 (m, 3H, **H_{arom}**), 7.63 (m, 2H, **NH**), for details see Syntheses and Characterizations

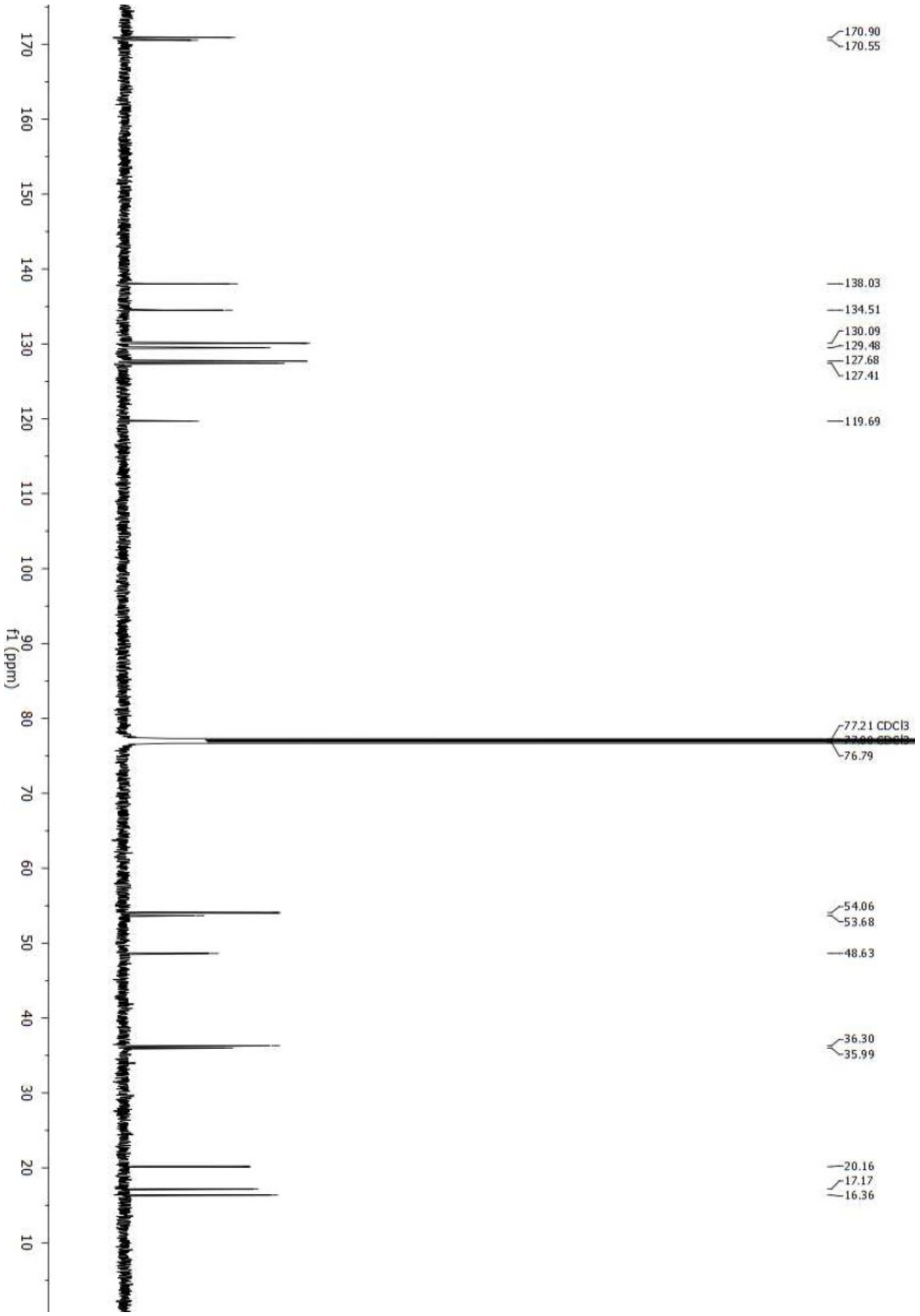

Fig. S19. ^{13}C NMR spectrum (151 MHz, $CDCl_3$) of compound **5b**: δ 16.36 (***C*****H$_2$CH$_2$**), 17.17 (**CH$_2$*C*H$_2$**), 20.16 (***C*****(CH$_2$)$_2$**), 35.99 (**SCH$_2$**), 36.30 (**NHCH*C*H$_2$**), 48.63 (1C, **C1**), 53.68 (1C, **C2**), 54.06 (**NH*C*HCO**), 119.69 (**CN**), 127.41 (***C*****$_{arom}$**), 127.68 (***C*****$_{arom}$**), 129.48 (*C*$_{arom}$), 130.09 (**C$_{arom}$**), 134.51 (***C*****$_{arom}$**), 138.03 (***C*****$_{arom}$**), 170.55 (***C*****ONH**), 170.90 (***C*****ONH**), for details see Syntheses and Characterizations

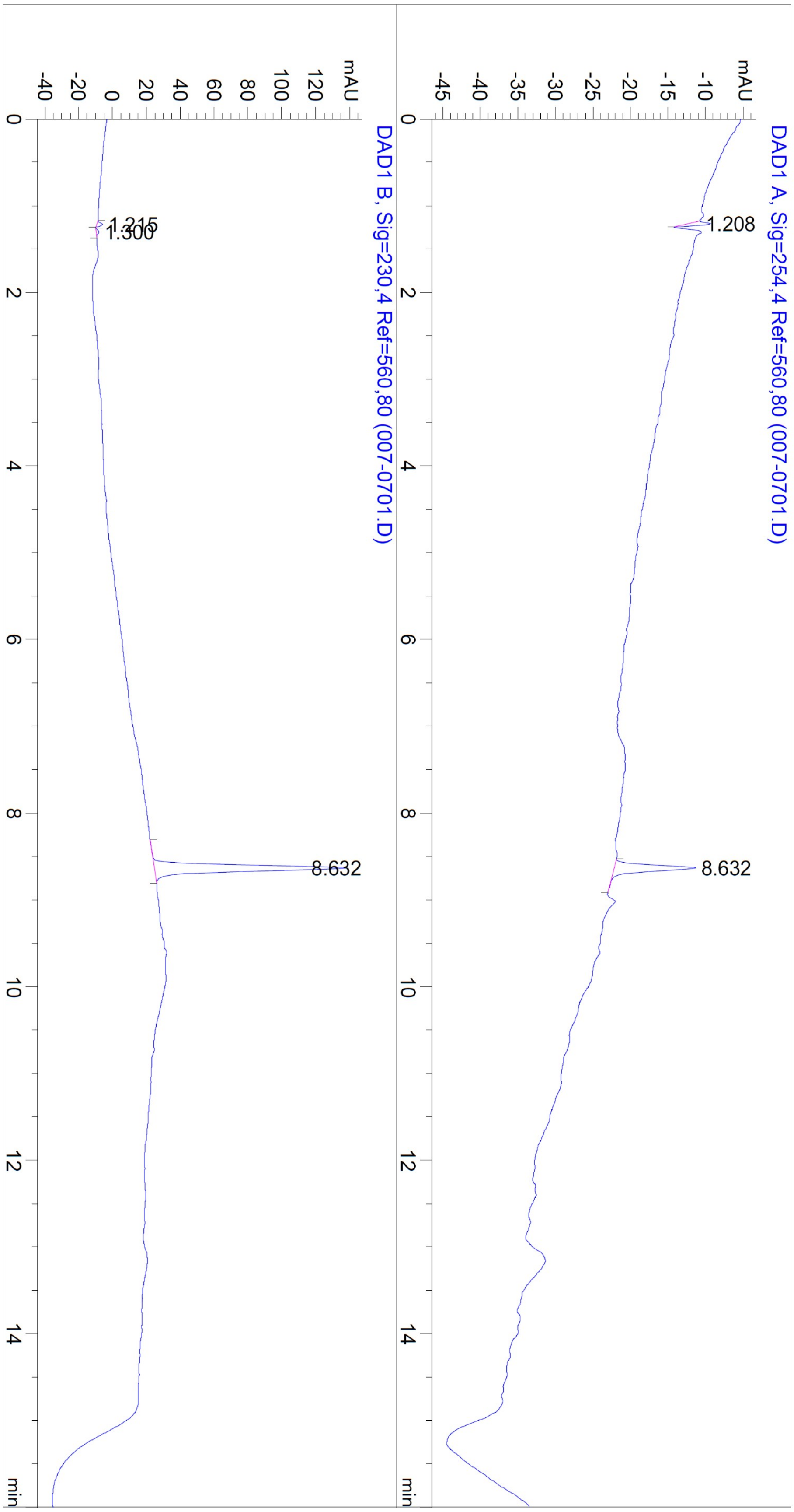

Fig. S20. HPLC chromatograms at 254 nm and 230 nm of compound **5b.** Peak at R_t 1.215 min: impurity; peak at R_t 8.632 min: compound **5b** with detected mass of 481.2 $[M+H]^+$, 98% purity.

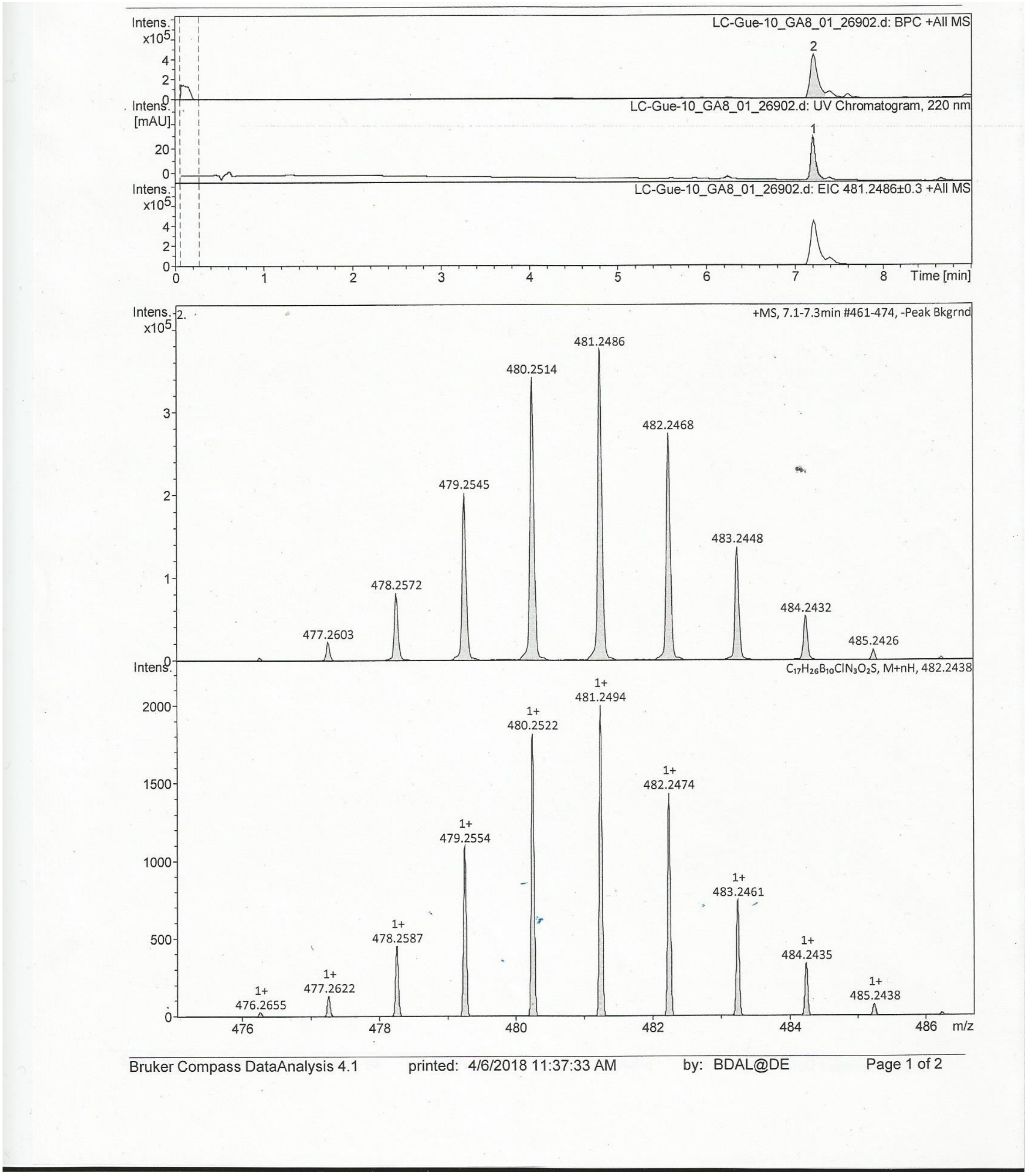

Fig. S21. HPLC-HRMS (micrOTOF-QIII) spectrum of compound **5b**; observed *m/z* corresponds to the expected molecular ions: $[M+H]^+$ = 481.2486 (100%). The characteristic isotopic pattern of the *closo*-1,2-$C_2B_{10}H_{12}$ carborane cage is clearly observable.

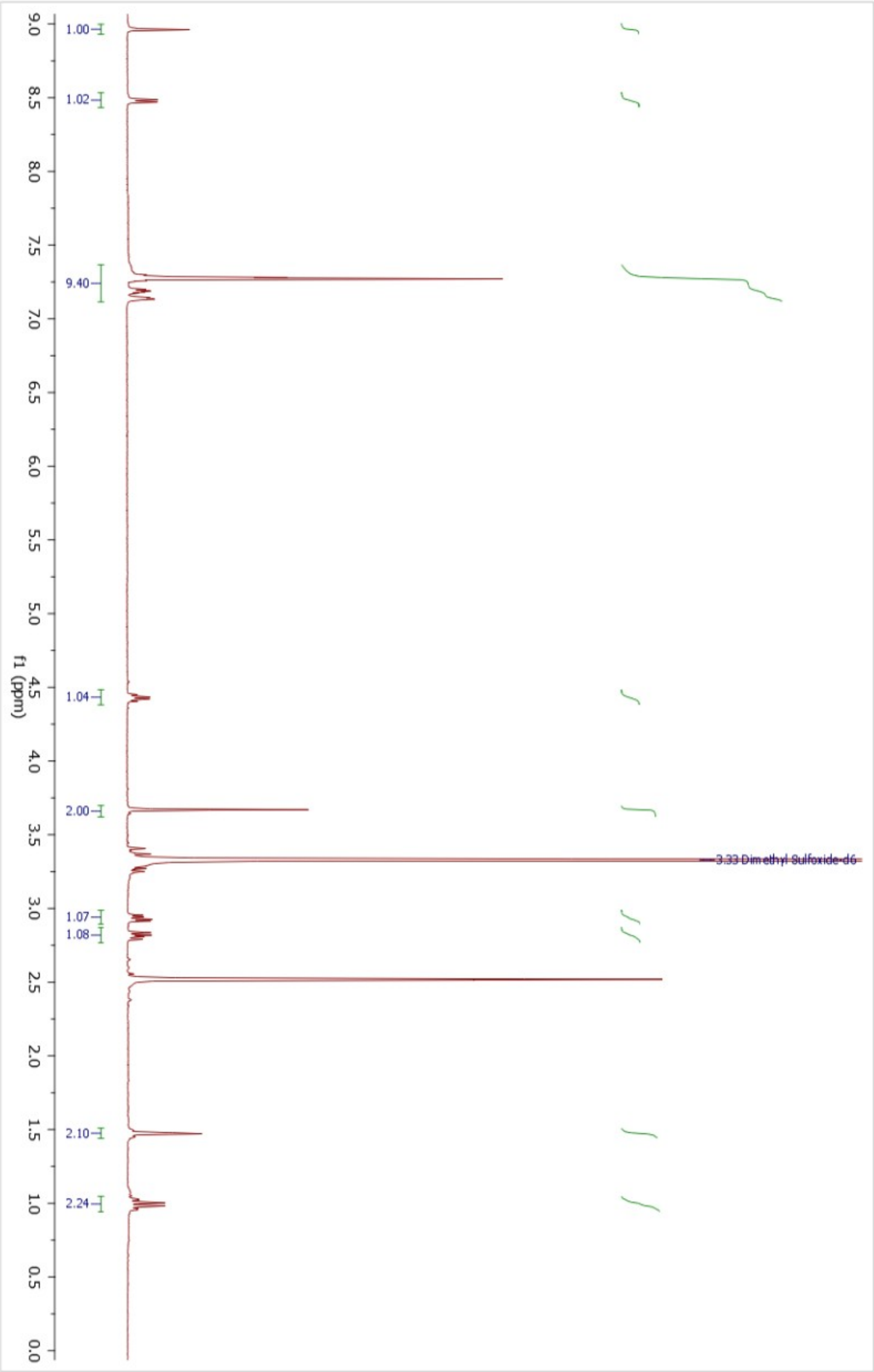

Fig. S22. ^{1}H NMR spectrum (500 MHz, $CDCl_3$) of compound **5a**: δ 0.93 – 1.00 (m, 2H, **CH_2CH_2)**, 1.42 – 1.47 (m, 2H, **CH_2CH_2**), 2.79 (dd, ^2J = 13.5 Hz, ^3J = 8.5 Hz, 1H, **-HCHC*H*$_2$**), 2.89 (dd, ^2J = 13.5 Hz, ^3J = 6.0 Hz, 1H, NHCHC*H*$_2$), 3.64 (s, 2H, SCH_2), 4.37 – 4.42 (m, 1H, NHC*H*CH_2), 7.09 – 7.17 (m, 2H, **H_{arom}**), 7.22 – 7.27 (m, 7H, **H_{arom}**), 8.45 (d, ^3J = 8.0 Hz, 1H, **CON*H*CH**), 8.93 (s, 1H, **CON*H*C(CH_2)$_2$**),), for details see Syntheses and Characterizations.

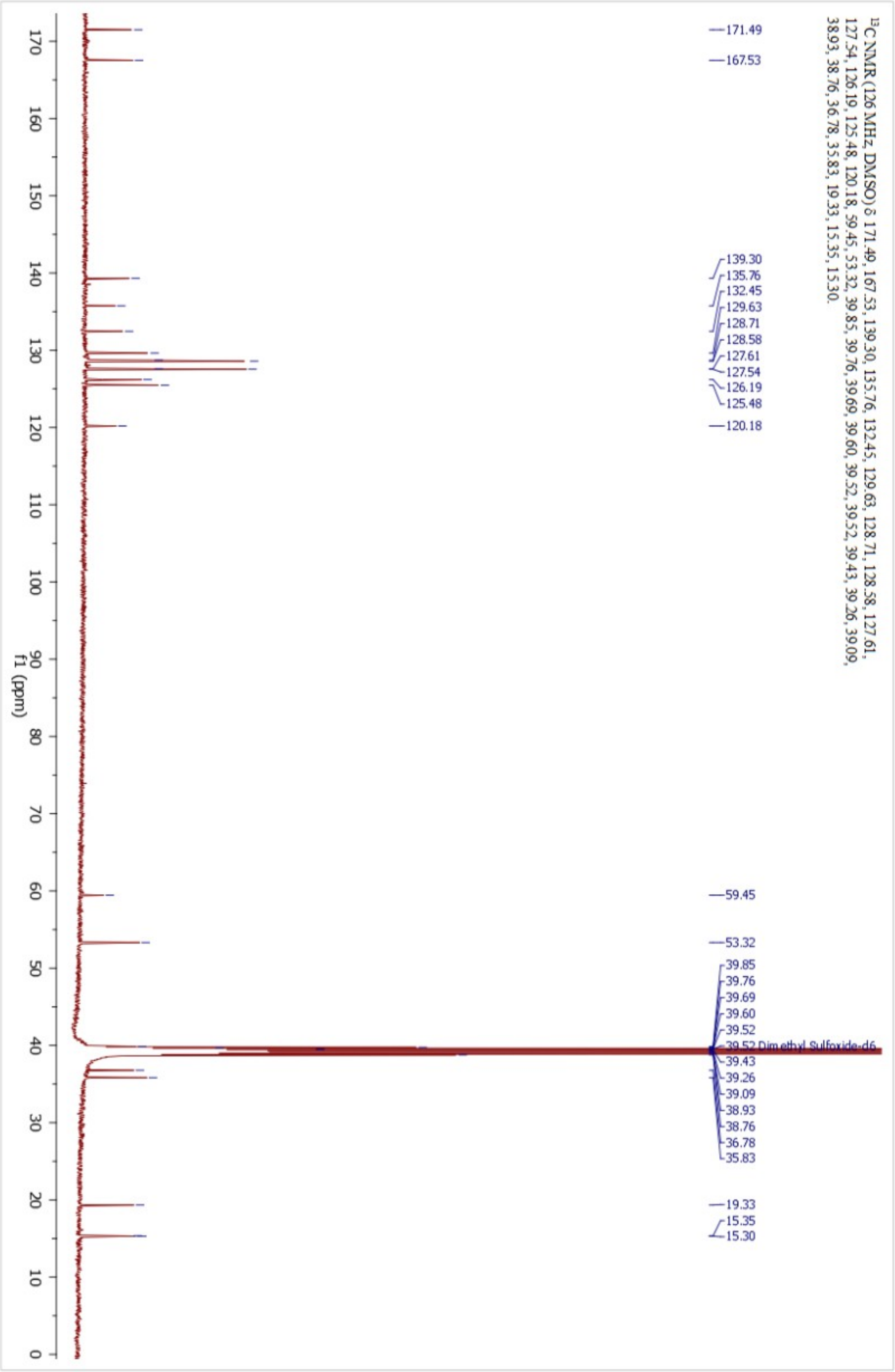

Fig. S23. ^{13}C NMR spectrum (151 MHz, $CDCl_3$) of compound **5a**: δ 15.30 (***C*H$_2$CH$_2$**), 15.35 (**CH$_2$*C*H$_2$**), 19.33 (***C*(CH$_2$)$_2$**), 35.83 (**S*C*H$_2$**), 36.78 (**NHCH*C*H$_2$**), 53.32 (**NH*C*HCO**), 120.18 (**CN**), 125.48 (***C*$_{arom}$**), 126.19 (***C*$_{arom}$**), 127.54 (***C*$_{arom}$**), 127.61 (***C*$_{arom}$**), 128.58 (***C*$_{arom}$**), 128.71 (***C*$_{arom}$**), 129.63 (***C*$_{arom}$**), 132.45 (***C*$_{arom}$**), 135.76 (***C*$_{arom}$**), 139.30 (***C*$_{arom}$**), 167.53 (***C*ONH**), for details see Syntheses and Characterizations.

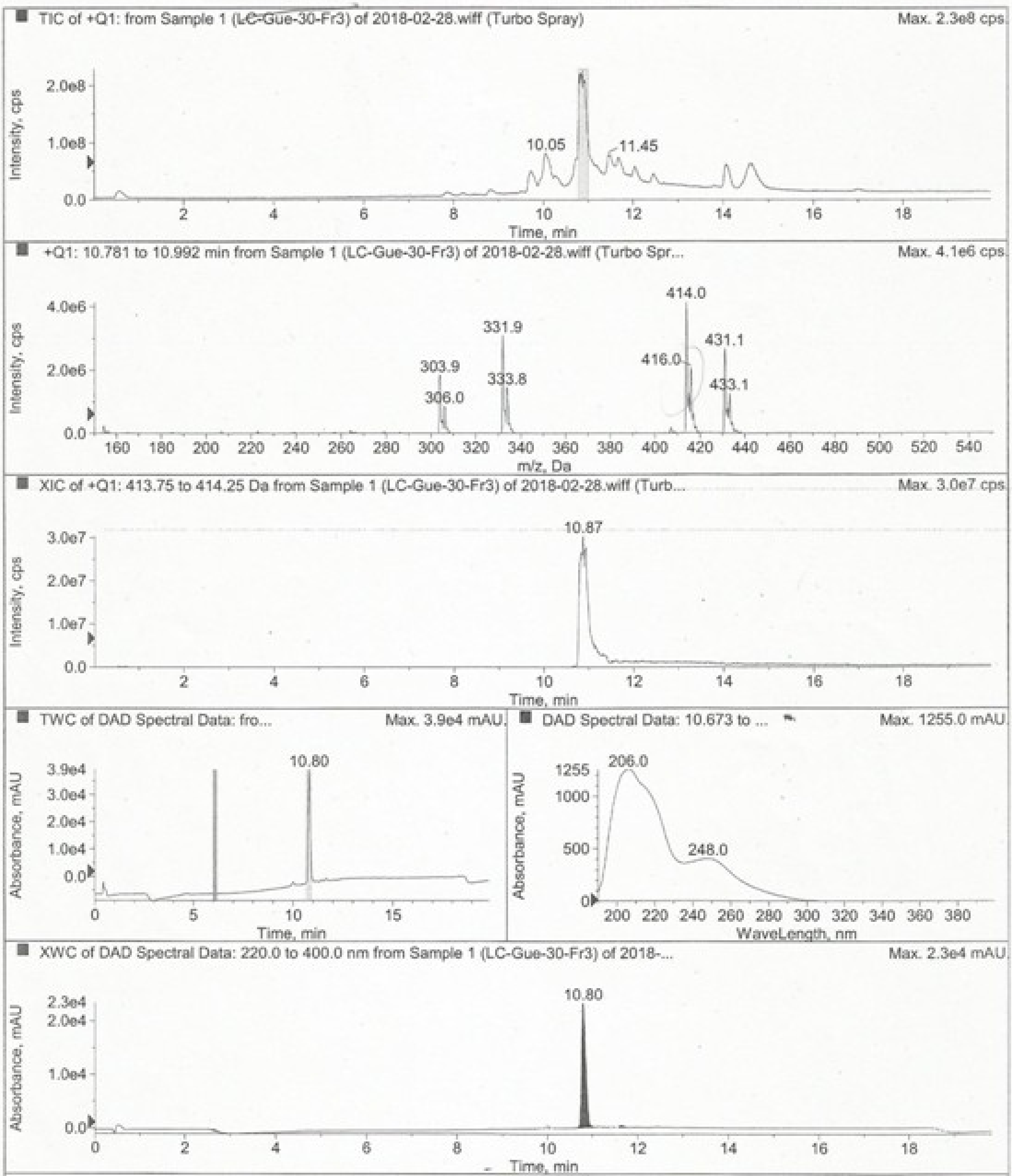

Peak List for "XWC of DAD Spectral Data: 220.0 to 400.0 nm from Sample 1 (LC-Gue-30-Fr3) of 2018-02-28.wiff"

	Time (min)	Area (mAU x min)	%Area	Height (mAU)	%Height	Width (min)	Baseline Type
1	2.5303	1690.2607	1.0470	7.8117	0.0320	0.2800	Base to Base
2	9.9985	4721.5379	2.9247	597.4262	2.4447	0.3933	Base to Base
3	10.7956	1.4957e5	92.6507	2.3276e4	95.2453	0.5667	Base to Base
4	11.4191	1094.9551	0.6783	188.5055	0.7714	0.2267	Base to Base
5	11.6391	2014.3725	1.2478	345.3450	1.4131	0.2267	Base to Base
6	18.5045	2343.2707	1.4515	22.8719	0.0936	0.3133	Base to Base

Fig. S24. HPLC–MS chromatogram of compound **5a**. Peak at R_t 10.80 min: compound **5a** with detected mass of 414.0 $[M+H]^+$.

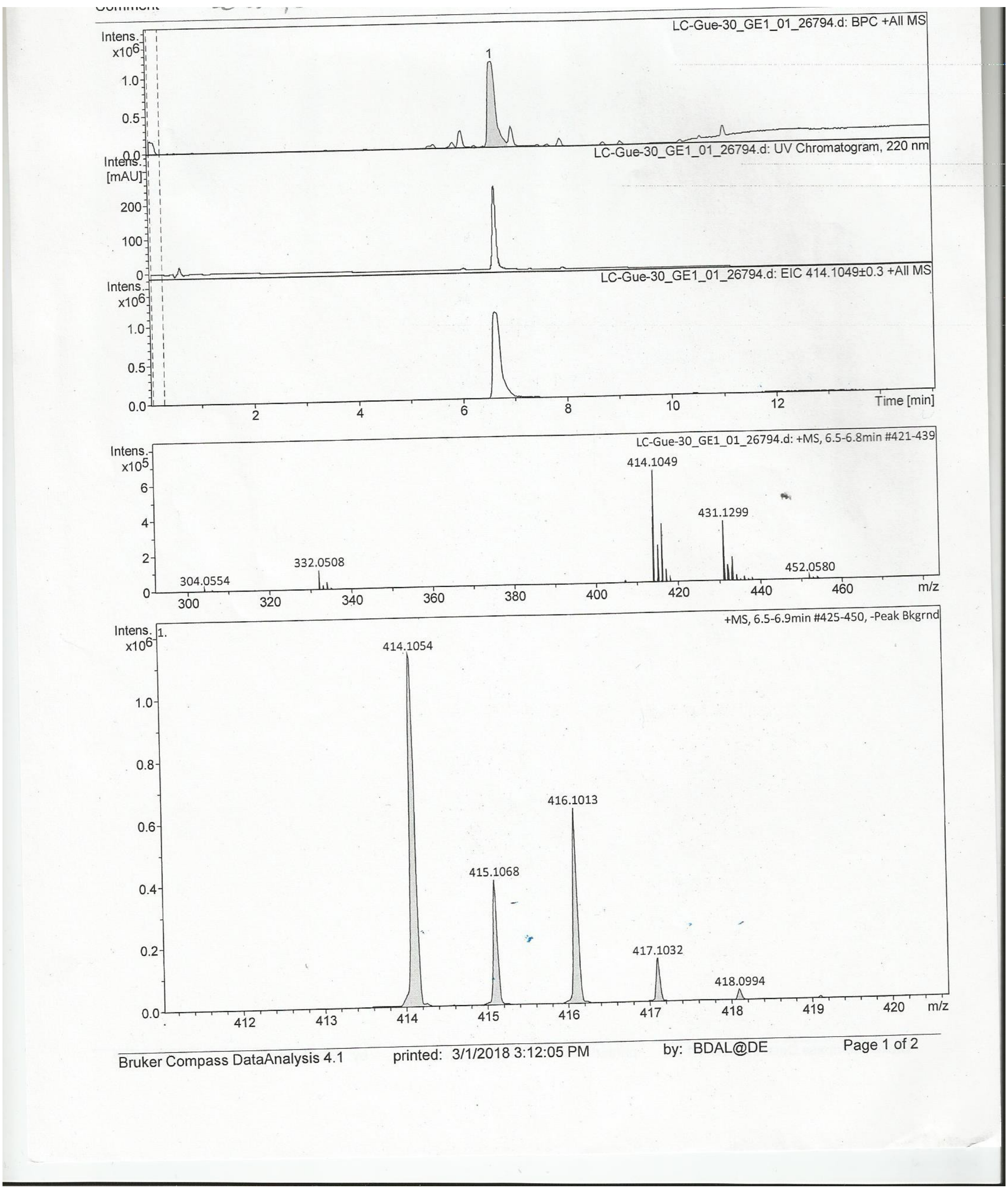

Fig. S25. HPLC-HRMS (micrOTOF-QIII) spectrum of compound **5a**; observed *m/z* corresponds to the expected molecular ions: $[M+H]^+$ = 414.1054 (100%). The characteristic isotopic pattern of chlorine is clearly observed.

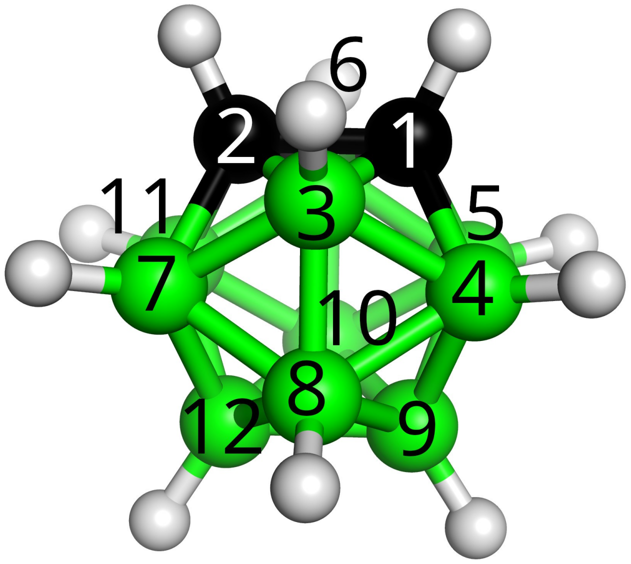

Fig. S26. The systematic vertex numbering of *closo*-1,2-$C_2B_{10}H_{12}$ (**1**).

References

1 J. Plešek and S. Heřmánek, *Chem. Ind.*, 1977, 360.

2 M. Giroud, B. Kuhn, S. Saint-Auret, C. Kuratli, R. E. Martin, F. Schuler, F. Diederich, M. Kaiser, R. Brun, T.Schirmeister and W. Haap, *J. Med. Chem.*, 2018, **61**, 3370–3388.

3 U. Mueller, R. Förster, M. Hellmig, F. U. Huschmann, A. Kastner, P. Malecki, S. Pühringer, M. Röwer, K. Sparta, M. Steffien, M. Ühlein, P. Wilk and M. S. Weiss, *Eur. Phys. J. Plus*, 2015, **130**, 141.

4 W. Kabsch, *Acta Crystallogr. D Biol. Crystallogr.*, 2010, **66**, 125–132.

5 A. Vagin and A. Teplyakov, *Acta Crystallogr. D Biol. Crystallogr.*, 2000, **56**, 1622–1624.

6 M. D. Winn, C. C. Ballard, K. D. Cowtan, E. J. Dodson, P. Emsley, P. R. Evans, R. M. Keegan, E. B. Krissinel, A. G. Leslie, A. McCoy, S. J. McNicholas, G. N. Murshudov, N. S. Pannu, E. A. Potterton, H. R. Powell, R. J. Read, A. Vagin and K. S. Wilson, *Acta Crystallogr. D Biol. Crystallogr.*, 2011, **67**, 235–242.

7 A. Jílková, M. Horn, P. Řezáčová, L. Marešová, P. Fajtová, J. Brynda, J. Vondrášek, J. H. McKerrow, C. R. Caffrey and M. Mareš, *Structure*, 2014, **22**, 1786–1798.

8 P. Emsley and K. Cowtan, *Acta Crystallogr. D Biol. Crystallogr.*, 2004, **60**, 2126–2132.

9 M. J. Betts and M. J. Sternberg, *Protein Eng.*, 1999, **12**, 271–283.

10 A. Klamt and G. Schuurmann, *J. Chem. Soc. Perkin Trans. 2*, 1993, 799–805.

11 R. Ahlrichs, M. Bar, M. Haser, H. Horn and C. Kolmel, *Chem. Phys. Lett.*, **1989**, *162*, 165–169.

12 J. Řezáč, *J. Comput. Chem.*, 2016, **37**, 1230–1237.

13 R. A. Nicholls, R. P. Joosten, F. Long, M. Wojdyr, A. Lebedev, E. Krissinel, L. Catapano, M. Fischer, P. Emsley and G. N. Murshudov, *Acta Crystallogr. D Biol. Crystallogr.*, 2021, **77**, 712–726.

14 A. A. Lebedev, P. Young, M. N. Isupov, O. V. Moroz, A. A. Vagin and G. N. Murshudov, *Acta Crystallogr. D Biol. Crystallogr*, 2012, **68**, 431–440.

15 S. C. Lovell, I. W. Davis, W. B. Arendall, III, P. I. de Bakker, J. M. Word, M. G. Prisant, J. S. Richardson and D. C. Richardson, *Proteins: Struct. Funct. Bioinform.*, 2003, **50**, 437–450.

16 H. J. Wiggers, J. R. Rocha, W. B. Fernandes, R. Sesti-Costa, Z. A. Carneiro, J. Cheleski, A. B. da Silva, L. Juliano, M. H. Cezari, J. S. Silva, J. H. McKerrow and C. A. Montanari, *PLoS Negl. Trop. Dis.*, 2013, **7**, e2370.

17 L. Cianni, C. Lemke, E. Gilberg, C. Feldmann, F Rosini, F. D. Rocho, J. F. Ribeiro, D. Y. Tezuka, C. D. Lopes, S. de Albuquerque, J. Bajorath, S. Laufer, A. Leitão, M. Gütschow and C. A. Montanari, *PLoS Negl. Trop. Dis.*, 2020, **14**, e0007755.

18 C. Lemke, L. Cianni, C. Feldmann, E. Gilberg, J. Yin, F. dos Reis Rocho, D. de Vita, U. Bartz, J. Bajorath, C. A. Montanari and M. Gütschow, *Bioorg. Med. Chem. Lett.*, 2020, **30**, 127420.

19 M. D. Mertens, J. Schmitz, m. Horn, N. Furtmann, J. Bajorath, M. Mareš and M. Gütschow, *ChemBioChem*, 2014, **15**, 955–959.

20 M. Frizler, F. Lohr, M. Lülsdorff and M. Gütschow, *Chem. Eur. J.*, 2011, **17**, 11419–11423.

21 A. T. Brünger, *Nature*, 1992, **355**, 472-475.

22 M. H. Abdulla, D. S. Ruelas, B. Wolff, J. Snedecor, K. C. Lim, F. Xu, A. R. Renslo, J. Williams, J. H. McKerrow and C. R. Caffrey, *PLoS Negl. Trop. Dis.*, 2009, **3**, e478.

23 T. Long, R. J. Neitz, R. Beasley, C. Kalyanaraman, B. M. Suzuki, M. P. Jacobson, C. Dissous, J. H. McKerrow, D. H. Drewry, W. J. Zuercher, R. Singh and C. R.Caffrey, *PLoS Negl. Trop. Dis*., 2016, **10**, e0004356.

24 A. Jílková, M. Horn, J. Fanfrlík, J. Küppers, P. Pachl, P. Řezáčová, M. Lepšík, P. Fajtová, P. Rubešová, M. Chanová, C. R. Caffrey, M. Gütschow and M. Mareš, *ACS Infect. Dis.*, 2021, **7**, 189-201.

25 A. Jílková, P. Rubešová, J. Fanfrlík, P. Fajtová, P. Řezáčová, J. Brynda, M. Lepšík, H. Mertlíková-Kaiserová, C. D. Emal, A. R. Renslo, W. R. Roush, M. Horn, C. R. Caffrey and M. Mareš, *ACS Infect. Dis.*, 2021, **7,** 1077-1088.

26 A. Tokarenko, B. Lišková, S. Smolen, N. Táborská, M. Tichý, S. Gurska, P. Perlíková, I. Frydrych, E. Tloušt'ová, P. Znojek, H. Mertlíková-Kaiserová, L Poštová Slavětínská, R. Pohl, B. Klepetářová, N. U. Khalid, Y. Wenren, R. R. Laposa, P. Dzubak, M. Hajduch and M. Hocek, *J. Med. Chem.*, 2018, **61**, 9347–9359.

27 A. Welch, *Crystals*, 2017, **7**, 234.
